# Supplementary material for: Consumption responses to an unconditional child allowance in the United States
Source: Nat Hum Behav. 2024 Feb 19;8(4):657–67. doi: 10.1038/s41562-024-01835-6 (PMC11045438; doi:10.1038/s41562-024-01835-6)
Supplement: Supplementary file 1 — Supplementary Appendices A–G. [file 41562_2024_1835_MOESM1_ESM.pdf]

nature human behaviour

Article https://doi.org/10.1038/s41562-024-01835-6

Consumption responses to an unconditional child allowance in the United States

In the format provided by the authors and unedited

Consumption Responses to an Unconditional Child Allowance in the United States

SUPPLEMENTARY MATERIALS

CONTENTS:

Appendix A: Institutional Details on the 2021 Expansion of the Child Tax Credit

Appendix B: Validation of Poverty Rate as Treatment Identifier & Details on CTC Simulation

Tables B1-B3
Figures B1-B2

Appendix C: Further Details on SafeGraph Data

Appendix D: Estimation Results with Continuous Treatment

Figures D1-D3

Appendix E: Measurement Validation

Figures E1-E6

Appendix F: Identification Tests

Figures F1-F4

Appendix G: Robustness Checks

Figures G1-G13

Tables G1–G7

Appendix H: Classification of Poverty Bins across Counties

Figures H1-H2

Appendix I: North American Industry Classification System (NAICS) Codes

Table I1

Appendix J: Disaggregated Results for Visits at Automobile, Clothing, Hobby and Sporting Goods, and Home Product Stores

Figure J1

APPENDIX A: Institutional Details on the 2021 Expansion of the Child Tax Credit

The American Rescue Plan Act of 2021 included the expansion of the Child Tax Credit (CTC) that this study investigates. This Appendix outlines key differences in the policy after the expansion, largely following the information provided in Crandall-Hollick (2021).

Prior Law: Prior to 2021, eligible taxpayers could use the CTC to reduce their federal tax liability by up to $2,000 per dependent child under 17 years old. The benefit value began to phase out at a 5 percent rate at modified adjusted gross incomes (AGI) above $200,000 (or $400,000 for married couples filing their tax returns jointly). If the tax filer's liabilities were less than the maximum value of the CTC, the tax filer could potentially receive the remainder of the CTC balance as a "refund" or direct payment at tax time. The maximum refund value was $1,400 per child under age 17. The refundable portion was only available for tax filers with earnings above $2,500.

2021 Changes: The primary changes under the American Rescue Plan Act include:

- **Full refundability**: The earnings requirement to receive the refundable portion of the CTC was eliminated. As a result, the full value of the CTC is refundable for all eligible units, including those reporting no or little earnings.
- **Age of eligibility**: 17-year-old children were eligible and counted as a dependent child for the tax unit.
- **Benefit values:** The maximum amount of the CTC benefit increased from $2,000 per child to $3,600 per child under age 6, and to $3,000 per child between the ages of 6 to 17.
- **Phase out:** The maximum benefit phased out at a 5 percent rate, for modified AGIs above $75,000 for single filers, $112,500 for head of household filers, and $150,000 for married joint filers. This first phase-out applied until the benefit reached $2,000, the maximum benefit level under prior law. A second phase-out applied for modified AGIs above $200,000 (or $400,000 for married couples filing their tax returns jointly). This second phase-out reduced, down to zero, the CTC by $50 for each $1,000 (5 percent rate).
- **Distribution of payments:** Half of the CTC value was paid in monthly installments between July and December 2021, while the other half was provided as a lump-sum payment at tax time.

APPENDIX B: Validation of Poverty Rate as Treatment Identifier & Details on CTC Simulation

We document here that our primary treatment identifier – a county's poverty rate – serves as a strong proxy for gains from the expansion of the CTC.

We simulate the increase in family income due to the CTC expansion using data from the 2019 Annual Social and Economic Supplement to the Current Population Survey (CPS-ASEC). To simulate this policy change, we first identify all individuals in the CPS-ASEC with dependents under the age of 18 and then calculate their benefit value under the CTC expansion according to the parameters described in Appendix A. Our simulation procedure accounts for both the monthly and the lump-sum component of the expanded CTC. We then estimate the net income gains due to the expanded CTC by taking the difference between the CTC benefit amount reported by respondents in the 2019 CPS-ASEC microdata and the expanded CTC benefit value that we simulated.[1] Note that children without a Social Security Number (SSN) were not eligible for the CTC (either under prior law or the expansion), but the CPS-ASEC does not include indicators for individuals without SSNs, thus we did not make eligibility distinctions on this basis.

We produce two measures of the income gain due to the expanded CTC: (1) an absolute income gain, which is the dollar value of added benefits due to the expanded CTC payments, and (2) a relative income gain, which is the percent change in income due to the expanded CTC payments relative to pre-expanded-CTC income. Given that our unit of analysis in the spending data is the county, and our primary measure of treatment intensity is the county's pre-treatment poverty rank, we convert the unit-level gains in the CPS-ASEC to weighted means at the county level for each of the three poverty rankings (high, medium, and low). Table B1 provides descriptive statistics on the estimated absolute and relative CTC gains by county poverty status and family type.

Table B2 presents estimation results of the relationship between a county's poverty status and gains from the CTC expansion among the subset of counties available in the CPS-ASEC. We find that a 10 percentage point increase in the poverty rate is statistically significantly associated with a $302 – equivalently 1.8 percent – increase in annual income. Figure B1 complements these estimates by showing robust linear associations between a county's poverty rate and the average relative income gain from the CTC.

Table B3 documents the gains from the childless EITC expansion. The average household in high-poverty counties only experiences a $20 absolute income gain relative to the average household in low-poverty counties; this absolute income gain is around 6 percent of the gains that we measure for the expanded CTC. It is thus unlikely that the childless EITC expansion meaningfully affects consumption patterns across high-, medium-, and low-mobility counties.

[1] Note that in the case of families who received a CTC for their older dependents (age 18 and over), we removed this credit amount before determining the net gain in income from the expansion.

Table B1: Descriptive statistics of absolute and relative income gains due to the expanded CTC by poverty status of county and characteristics of tax unit

	**All Tax Units**		**Tax Units with Children**		**Tax Units with 2 Children**	
	Mean	Std. Dev.	Mean	Std. Dev.	Mean	Std. Dev.
**Absolute Gain in Income**						
Low Poverty Counties	1,374	2,299	2,472	2,184	2,454	1,536
Medium Pov. Counties	1,599	2,643	2,833	2,426	2,816	1,612
High Poverty Counties	1,682	2,597	3,013	2,380	2,991	1,623
**Relative Gain in Income**						
Low Poverty Counties	2.7%	9.6%	4.7%	11.8%	4.6%	12.4%
Medium Pov. Counties	3.9%	12.6%	6.9%	15.1%	6.2%	12.1%
High Poverty Counties	4.9%	13.2%	8.7%	17.1%	7.8%	13.0%

Note: This analysis is limited to counties observed in the CPS-ASEC. "Absolute Gain" refers to the additional dollar value of income due to the CTC expansion. "Relative Gain" refers to the percent increase in income. * $p < 0.05$, ** $p < 0.01$, *** $p < 0.001$. All P values are based on two-tailed tests. No adjustments were made for multiple comparisons.

Table B2: Association of county poverty status and gains from the CTC expansion

	Absolute $ Gain due to CTC Expansion	Log $ Gain due to CTC Expansion	Absolute $ Gain due to CTC Expansion	Log $ Gain due to CTC Expansion
Medium Poverty County X Post-CTC Expansion	124.34*** (24.40)	0.009*** (0.001)		
High Poverty County X Post-CTC Expansion	273.86*** (30.15)	0.018*** (0.001)		
County Poverty Rate X Post-CTC Expansion			2,622.40*** (197.80)	0.151*** (0.009)

Note: The models include state fixed effects to match the specification in our primary analysis. This analysis is limited to counties observed in the CPS-ASEC. "Absolute Gain" refers to the additional dollar value of income due to the CTC expansion. * $p < 0.05$, ** $p < 0.01$, *** $p < 0.001$. All P values are based on two-tailed tests. No adjustments were made for multiple comparisons.

Table B3: Association of county poverty status and gains from the childless EITC expansion

	Absolute $ Gain due to Childless EITC Expansion	Log $ Gain due to Childless EITC Expansion	Absolute $ Gain due to Childless EITC Expansion	Log $ Gain due to Childless EITC Expansion
Medium Poverty County X Post-Expansion	17.65*** (3.246)	0.001*** (0.000)		
High Poverty County X Post-Expansion	18.47*** (4.011)	0.001*** (0.000)		
Country Poverty Rate X Post-Expansion			180.21*** (26.327)	0.012*** (0.002)

Note: The models include state fixed effects to match the specification in our primary analysis. This analysis is limited to counties observed in the CPS-ASEC. "Absolute Gain" refers to the additional dollar value of income due to the childless EITC expansion. * $p < 0.05$, ** $p < 0.01$, *** $p < 0.001$. All P values are based on two-tailed tests. No adjustments were made for multiple comparisons.

Figure B1: Binned scatterplot of association of county poverty status and mean percent income gain from the expanded CTC

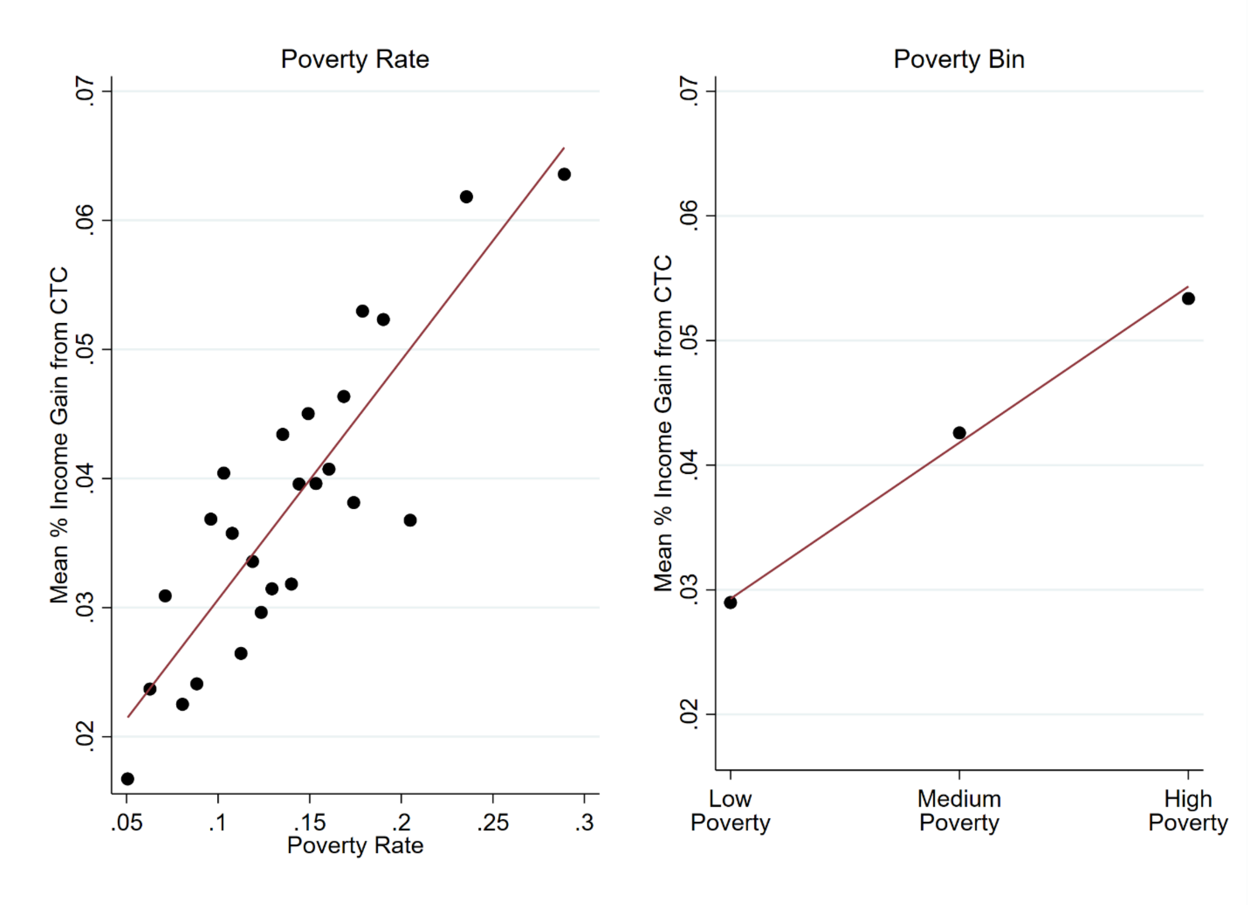

Note: Estimates are limited to the subset of counties available in the CPS-ASEC.

Figure B2: Difference in CTC take-up rate relative to low-poverty counties, by within county household income decile (2022)

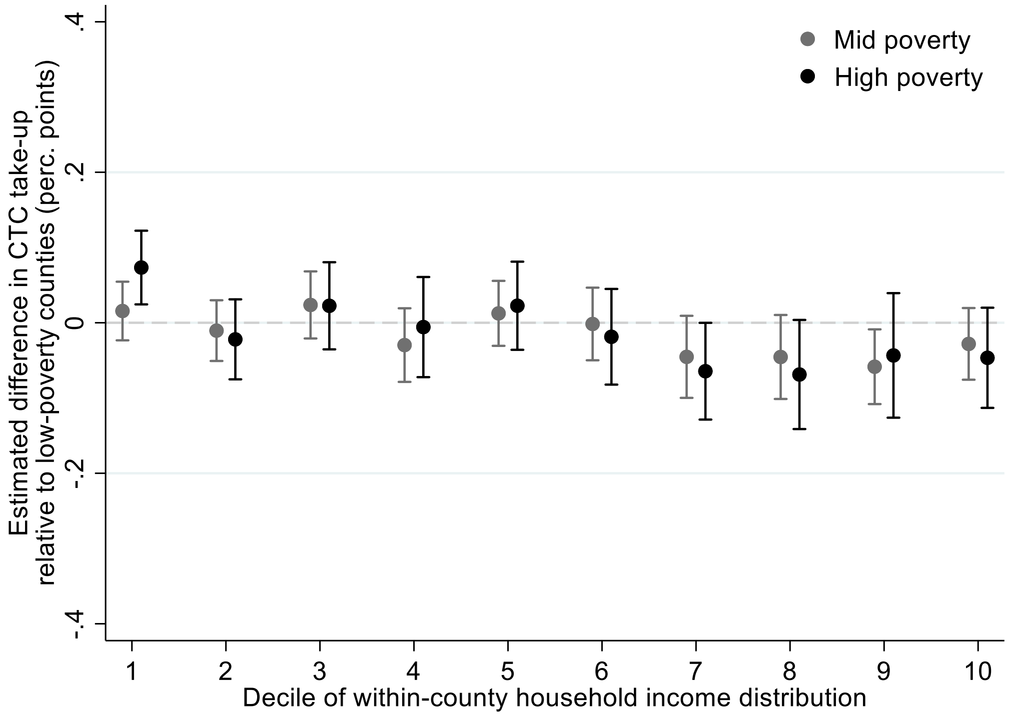

Note: Estimates are limited to the subset of counties available in the CPS-ASEC. The capped horizontal bars represent 95 percent confidence intervals. Sample size = 152,732.

Appendix C: Further Details on SafeGraph Data

SafeGraph provides various datasets on individual places of interest in the United States. Each place of interest is assigned a unique identifier called a *placekey*, which can be used to link places of interest across datasets. These places of interest include schools, retail stores, child care centers, restaurants, and other physical establishments.

In-Person Visits

To measure in-person visits, we use two datasets from SafeGraph: Places and Patterns. Places data includes location name, brand information (including 6-digit NAICS codes), and geographic information for each unique placekey. Patterns data includes aggregated, anonymized mobile-phone data on mobility patterns and foot traffic to each unique placekey. This information is provided by Global Positioning System (GPS) data from 10% of mobile devices (more than 40 million) in the U.S.

SafeGraph registers in-person visits as mobile phone entries into a given place of interest for four minutes or more. Although SafeGraph no longer provides Patterns data (as of 2023), historical patterns data can be obtained from Dewey Data, a platform that connects data vendors with academic researchers. Places data continues to be provided by SafeGraph.

Spending

To measure monetary spending, we use anonymized debit and credit card transaction data provided by SafeGraph's Spend data. Transaction information includes mean transaction size per month at individual places of interest in the U.S. Each place of interest is assigned a *placekey* that can easily be linked to other SafeGraph datasets.

APPENDIX D: Estimates with Continuous Treatment

Figure D1: Parallel Trends Assumption: Estimated time-trend of in-person visits and spending by establishment type in higher-poverty counties from January to June 2021 (prior to the introduction of monthly CTC payments)

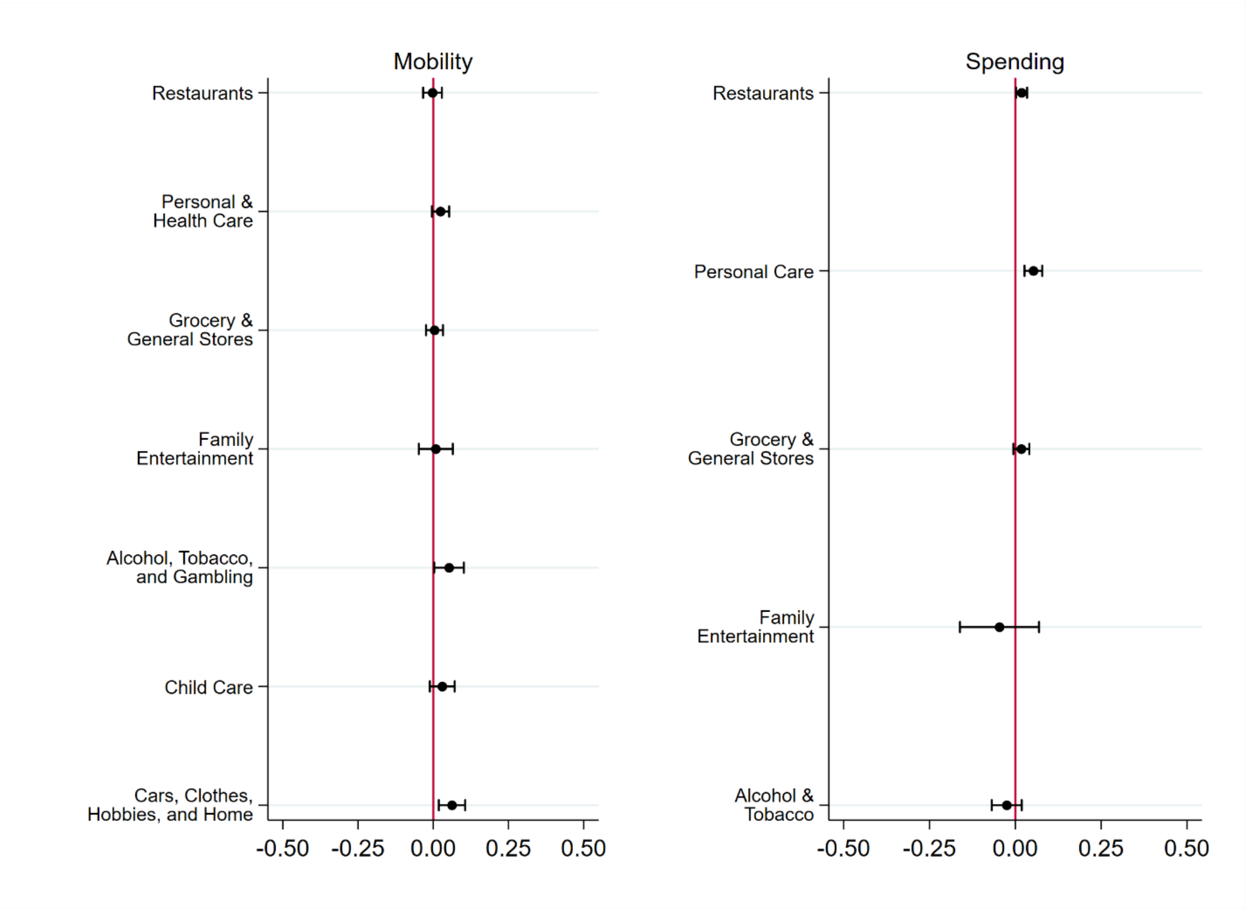

Note: Estimated effect of the interaction of monthly time trend and county poverty rate on in-person visits to and spending at different establishment types. Data is from January to June 2021. See Table 1 for a description of establishment types. The capped horizontal bars report 95 percent confidence intervals. For "Mobility," the sample sizes are 18,450 (Restaurants), 17,994 (Personal & Health Care), 18,492 (General & Grocery Stores), 14,406 (Family Entertainment), 16,458 (Alcohol, Tobacco, and Gambling), 16,890 (Child Care), and 18,054 (Cars, Clothes, Hobbies, and Home). For "Spending," the sample sizes are 16,644 (Restaurants), 11,742 (Personal Care), 17,640 (General & Grocery Stores), 6,318 (Family Entertainment), and 8,784 (Alcohol and Tobacco).

Figure D2: Effects of expanded CTC payments on seasonally-adjusted visits to establishment types using county-level poverty rate as a continuous treatment by payment type

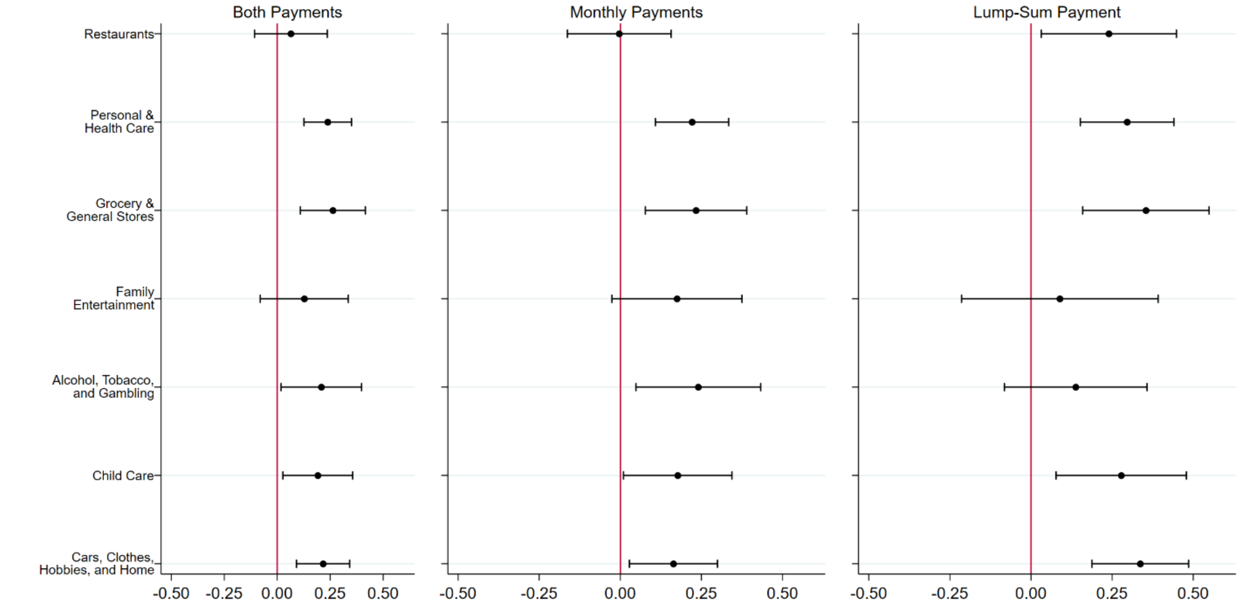

Note: Estimates of coefficient β_4 from model (1) using county-level poverty rate as a continuous treatment measure. See Table 1 for a description of establishment types. The capped horizontal bars represent 95 percent confidence intervals. "Monthly Payments" refers to the effects of the monthly-distributed CTC payments between July and December 2021. Sample sizes are 51,629 (Restaurants), 50,626 (Personal & Health Care), 51,799 (General & Grocery Stores), 40,579 (Family Entertainment), 46,155 (Alcohol, Tobacco, and Gambling), 47,464 (Child Care), and 50,643 (Cars, Clothes, Hobbies, and Home).

Figure D3: Effects of the expanded CTC payments on spending per transaction at establishment types using county-level poverty rate as a continuous treatment by payment type

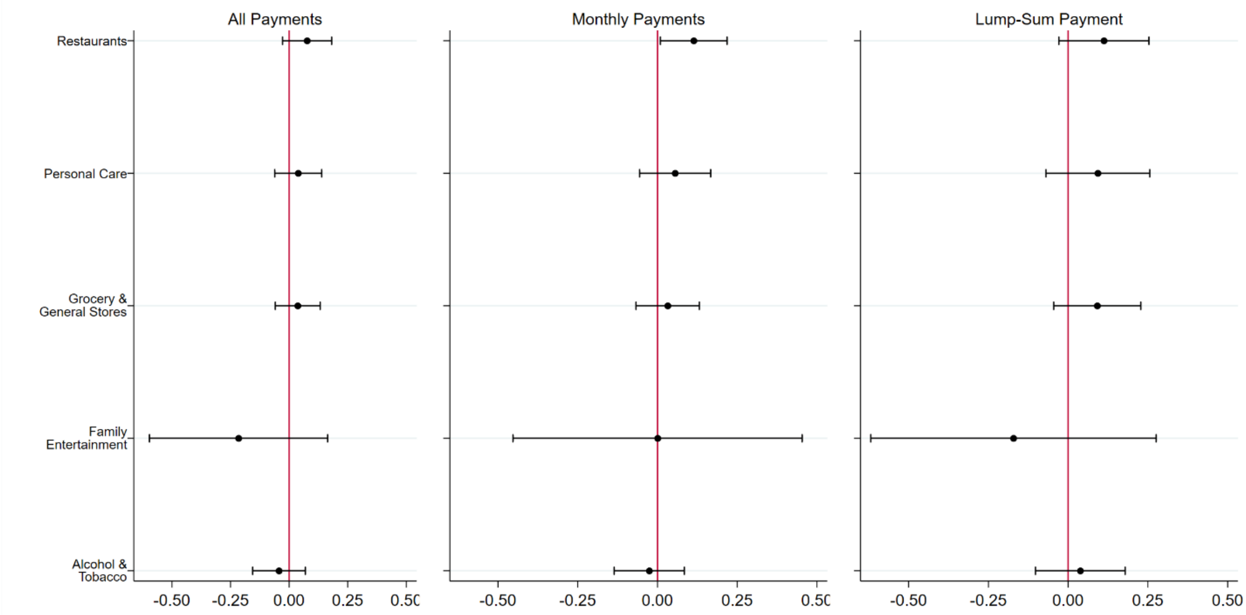

Note: Estimates of coefficient β_4 from model (1) using county-level poverty rate as continuous treatment measure. See Table 1 for a description of establishment types. The capped horizontal bars represent 95 percent confidence intervals. "Monthly Payments" refers to the effects of the monthly-distributed CTC payments between July and December 2021. "Lump-Sum Payment" refers to the effects of the single, tax-time CTC payment distributed in spring 2022. Sample sizes are 24,928 (Restaurants), 17,606 (Personal Care), 26,438 (General & Grocery Stores), 9,463 (Family Entertainment), and 13,169 (Alcohol and Tobacco).

APPENDIX E: Measurement Validation

Figure E1: Is mobility data a useful proxy for consumption on the extensive margin? Binned scatterplots of county-level, monthly in-person visits to establishments (X-axis) and transactions with debit/credit cards (Y-axis)

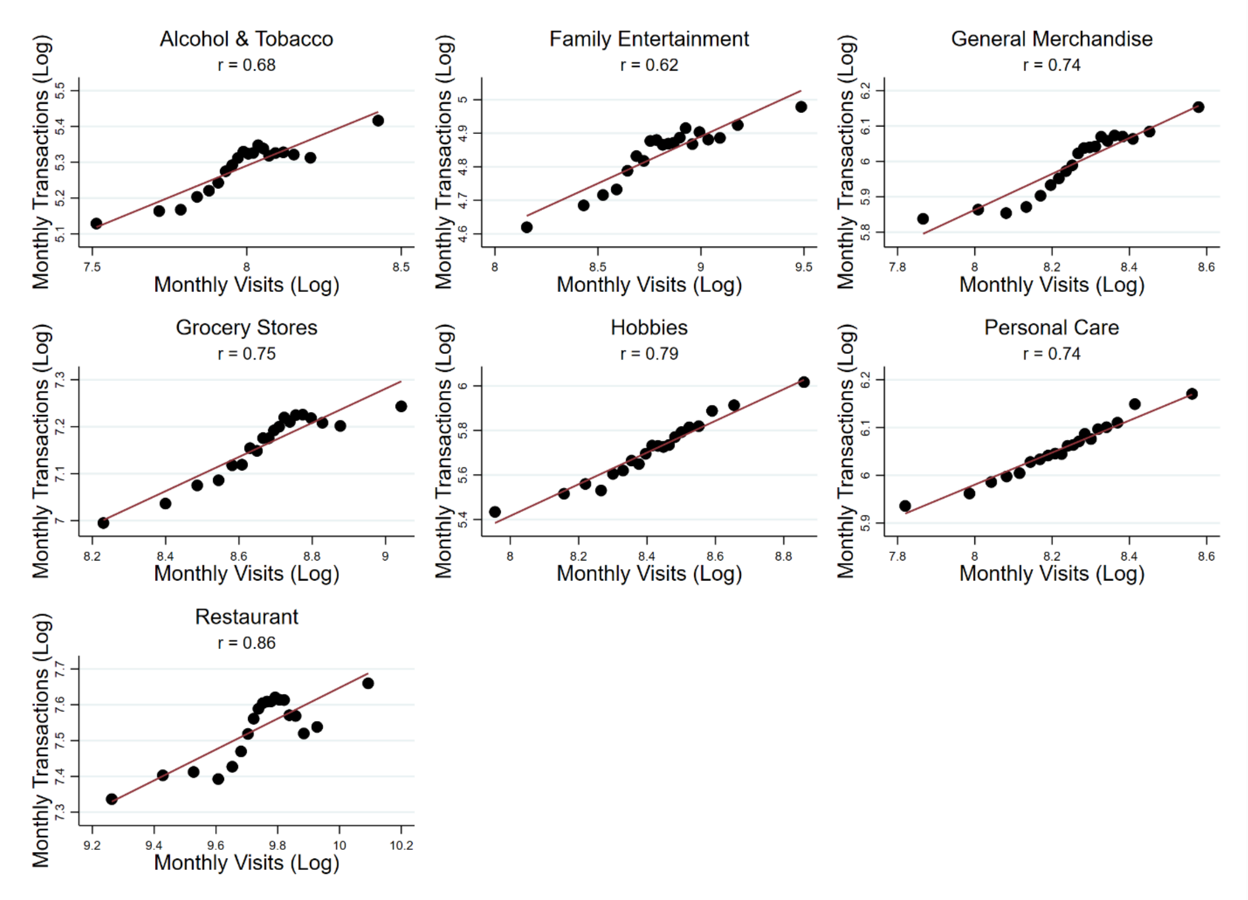

Note: The sample for this analysis is limited to counties for which data on both in-person visits and debit/credit card transactions is available. The total number of establishments per month included in the binned scatterplots ranges from 6,141 (Family Entertainment venues) to 213,028 (Restaurants). The figure presents a more disaggregated categorization of establishments (7 categories) than presented in our primary spending findings (5 categories) to ensure a closer one-to-one match with the related establishments for which we have mobility data.

Figure E2: Are the mobility data representative of the population distribution of U.S. counties? Binned scatterplots of Census population counts at county level (X-axis) and mean monthly in-person visits to establishments by county (Y-axis) by poverty status of county

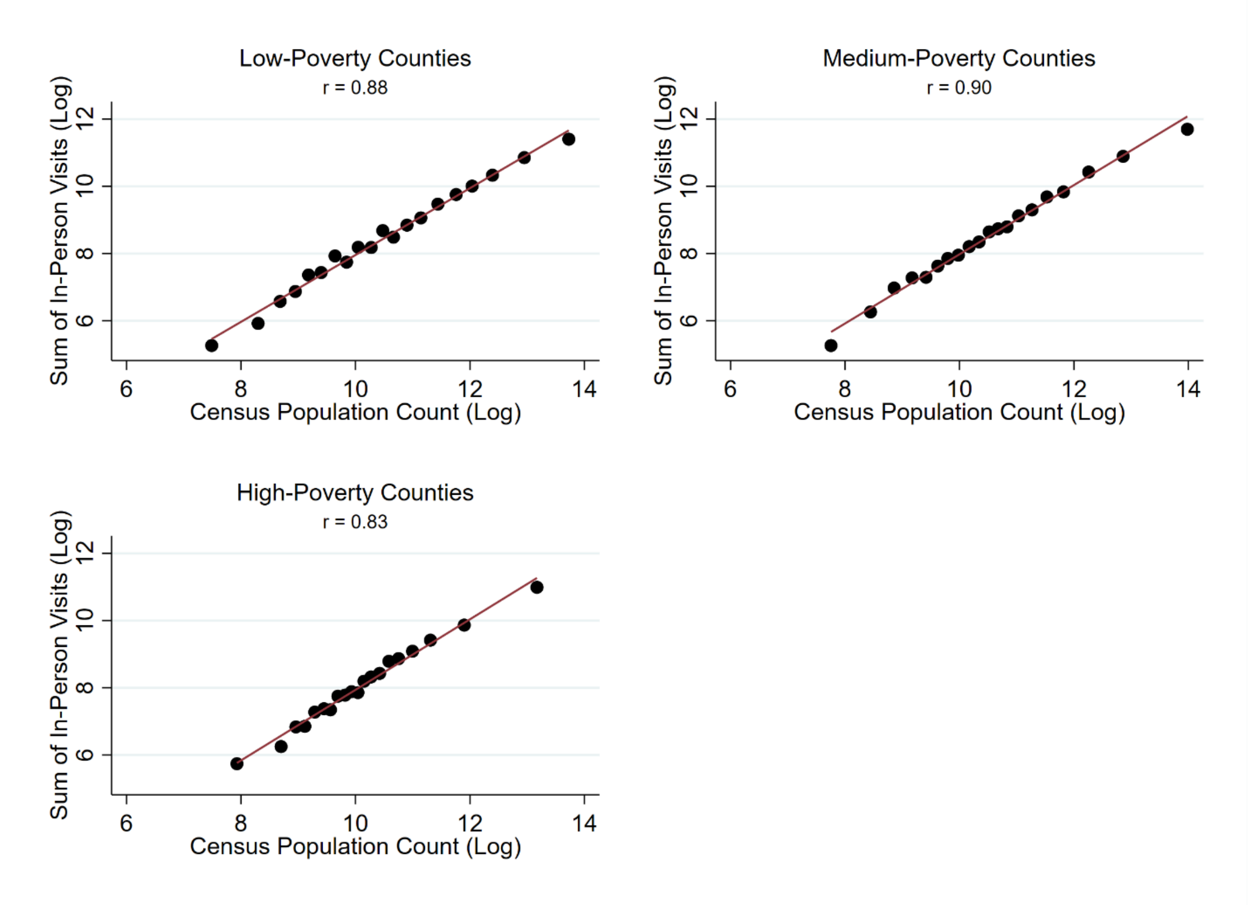

Note: The sum of in-person visits is the authors' calculations from SafeGraph data. The population counts are from the U.S. Census Bureau.

Figure E3: Binned scatterplot of the county-level relationship between smartphone ownership rate and poverty rate

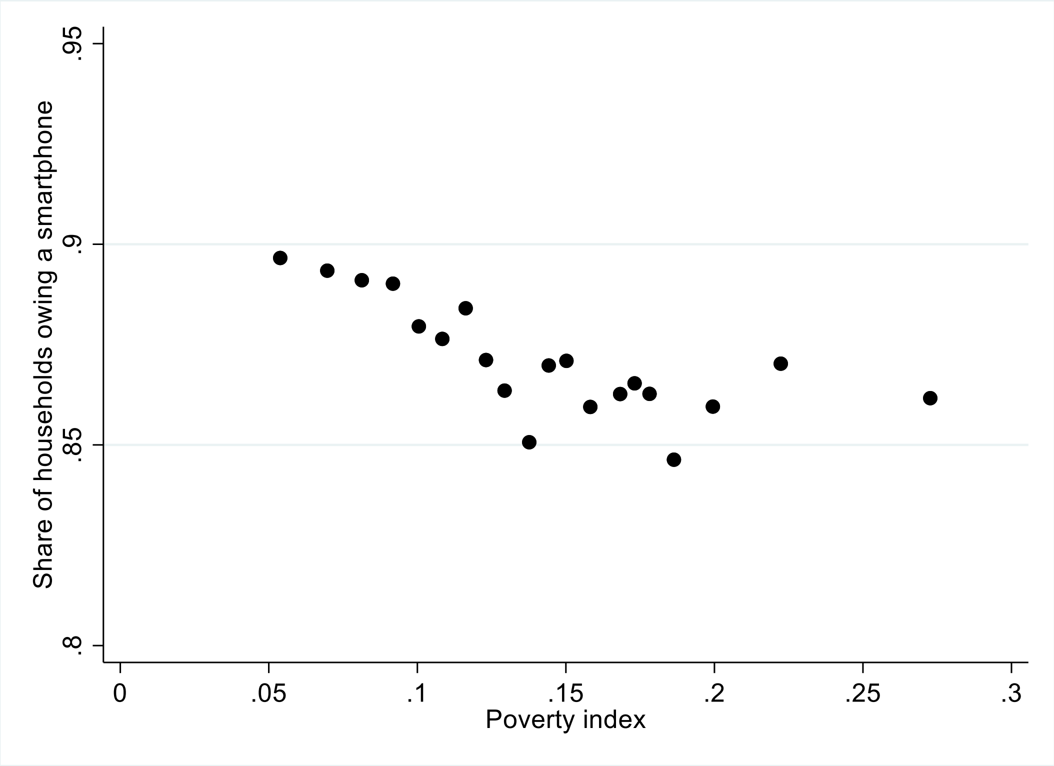

Note: Smartphone ownership data is from the 2019 American Community Survey. Sample of available counties.

Figure E4: Difference in smartphone ownership rate in high- and mid-poverty counties relative to low-poverty counties, by within county household income decile

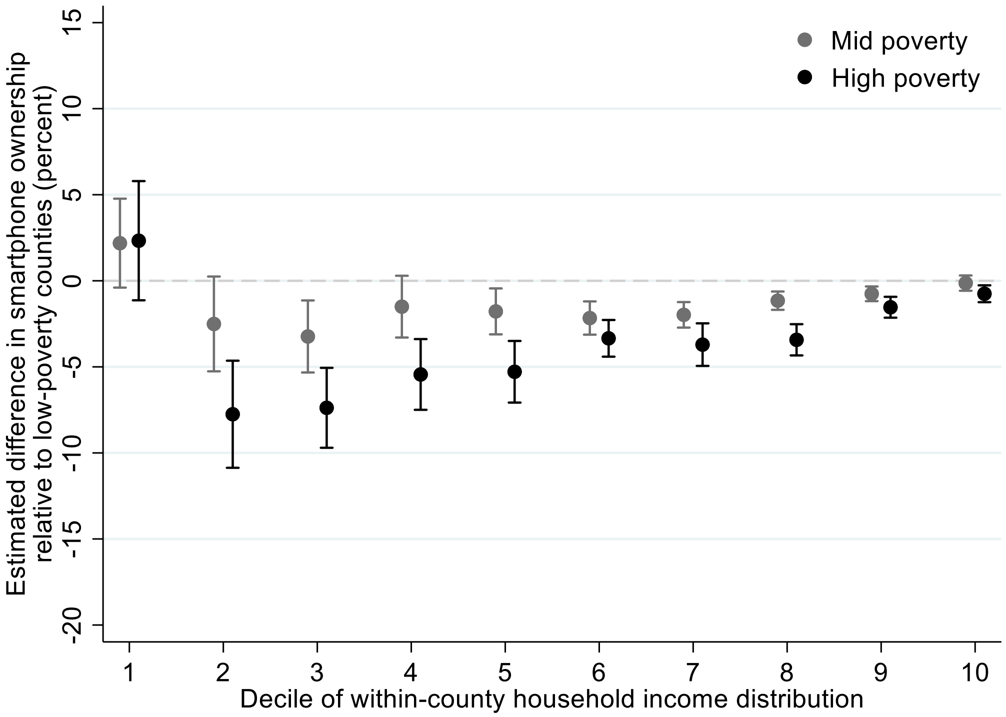

Note: Smartphone ownership data is from the 2019 American Community Survey. Sample of available counties. The capped horizontal bars represent 95 percent confidence intervals. Sample size = 1,548,188.

Figure E5: Falsification Test #1: Does the monthly CTC affect visits to schools in our analysis?

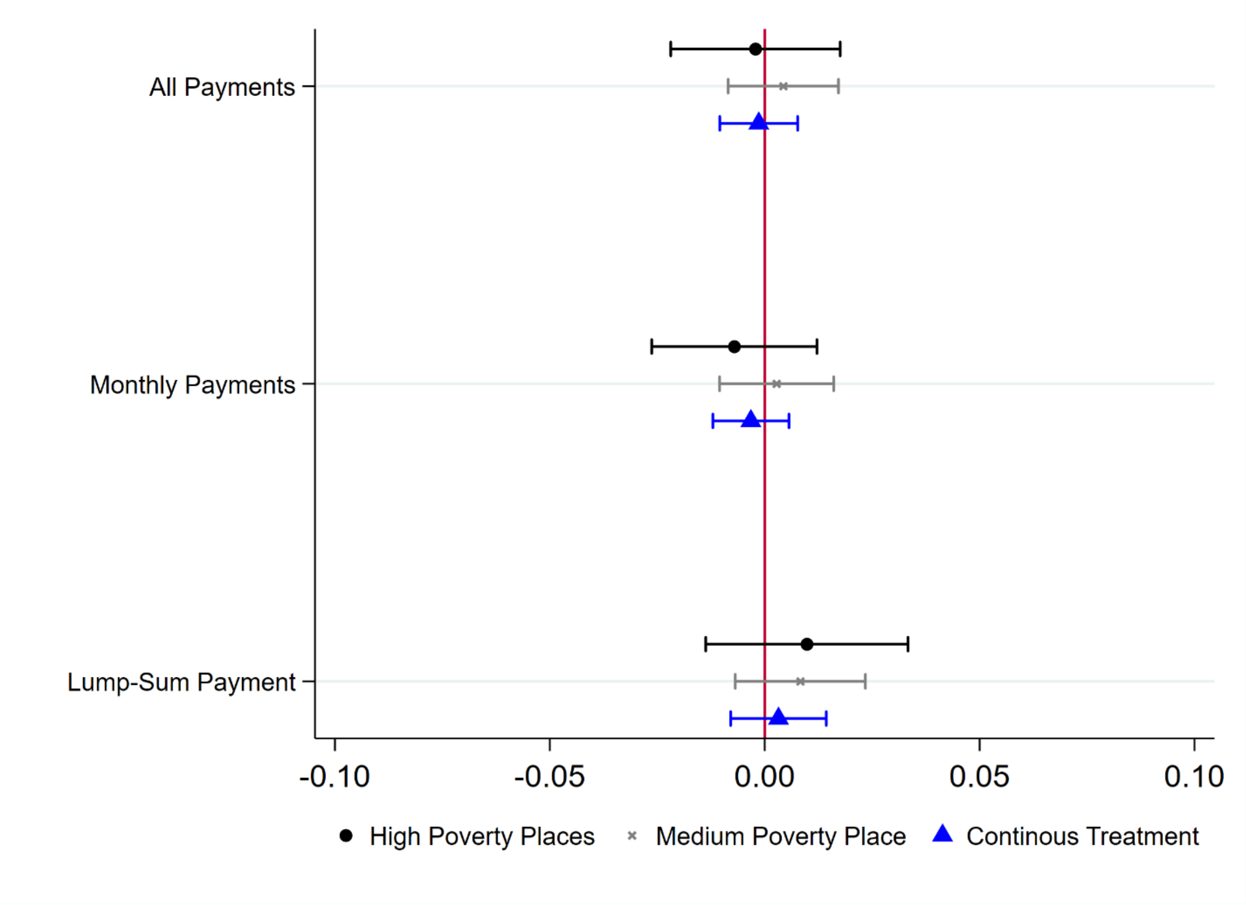

Note: Estimates of coefficient β_4 from model (1) for seasonally-adjusted changes to K-12 public schools. The capped horizontal bars represent 95 percent confidence intervals. "Monthly Payments" refers to the effects of the monthly-distributed CTC payments between July and December 2021. "Lump-Sum Payment" refers to the effects of the single, tax-time CTC payment distributed in spring 2022. The continuous treatment is standardized in this figure for comparability with point estimates from poverty bin analyses. Sample size: 52,895.

Figure E6: Falsification Test #2: Does the monthly CTC affect visits to religious institutions in our analysis?

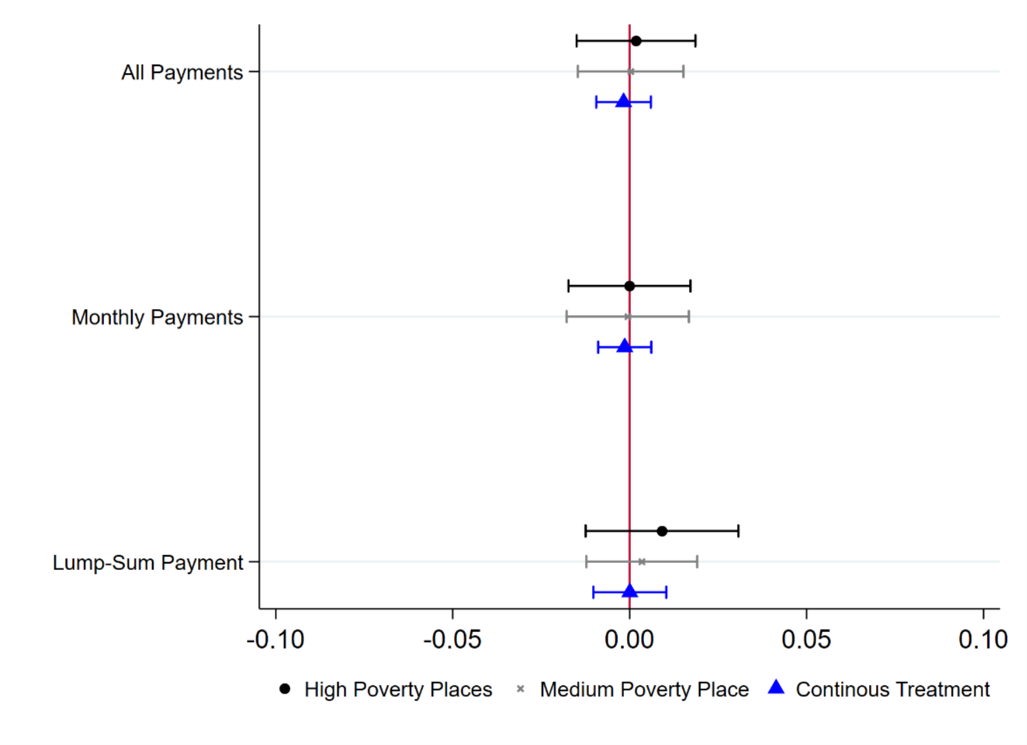

Note: Estimates of coefficient β_4 from model (1) for seasonally-adjusted changes to religious institutions (places of worship). The capped horizontal bars represent 95 percent confidence intervals. "Monthly Payments" refers to the effects of the monthly-distributed CTC payments between July and December 2021. "Lump-Sum Payment" refers to the effects of the single, tax-time CTC payment distributed in spring 2022. The continuous treatment is standardized in this figure for comparability with point estimates from poverty bin analyses. Sample size: 53,261.

APPENDIX F: Identification Tests

Figure F1: Parallel Trends Assumption: Estimated time-trend of in-person visits and spending in high- and medium-poverty counties relative to low-poverty counties by establishment type from January to June 2021 (prior to the introduction of monthly CTC payments)

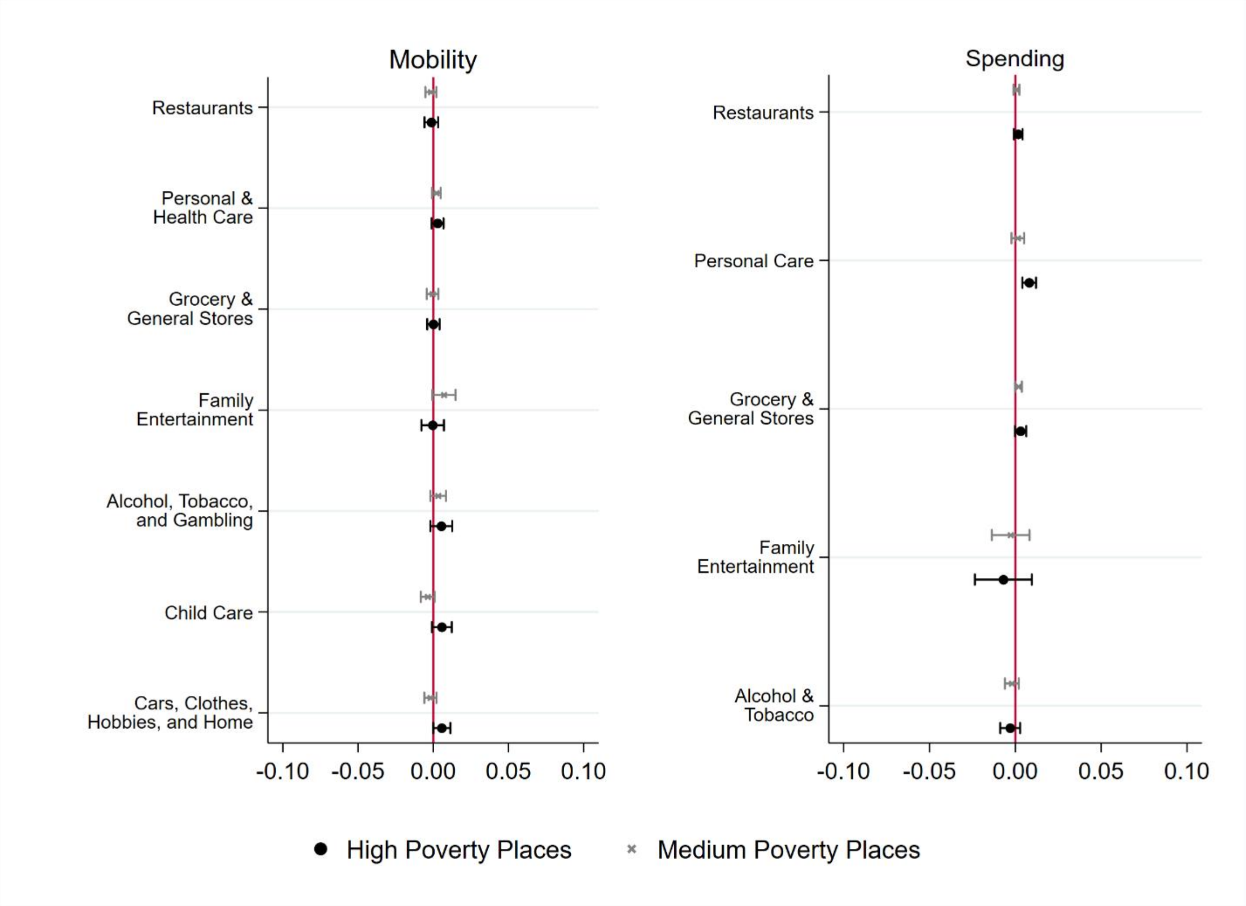

Note: Estimated monthly time trend on in-person visits to and spending at different establishment types by county poverty status relative to low-poverty counties. Data is from January to June 2021. See Table 1 for a description of establishment types. The capped horizontal bars report 95 percent confidence intervals. For "Mobility," the sample sizes are 18,450 (Restaurants), 17,994 (Personal & Health Care), 18,492 (General & Grocery Stores), 14,406 (Family Entertainment), 16,458 (Alcohol, Tobacco, and Gambling), 16,890 (Child Care), and 18,054 (Cars, Clothes, Hobbies, and Home). For "Spending," the sample sizes are 16,644 (Restaurants), 11,742 (Personal Care), 17,640 (General & Grocery Stores), 6,318 (Family Entertainment), and 8,784 (Alcohol and Tobacco).

Figure F2: Extended event study estimates of the effect of expanded CTC on in-person visits to different establishment types in high-poverty counties relative to low-poverty counties (pre-treatment period beginning in July 2020)

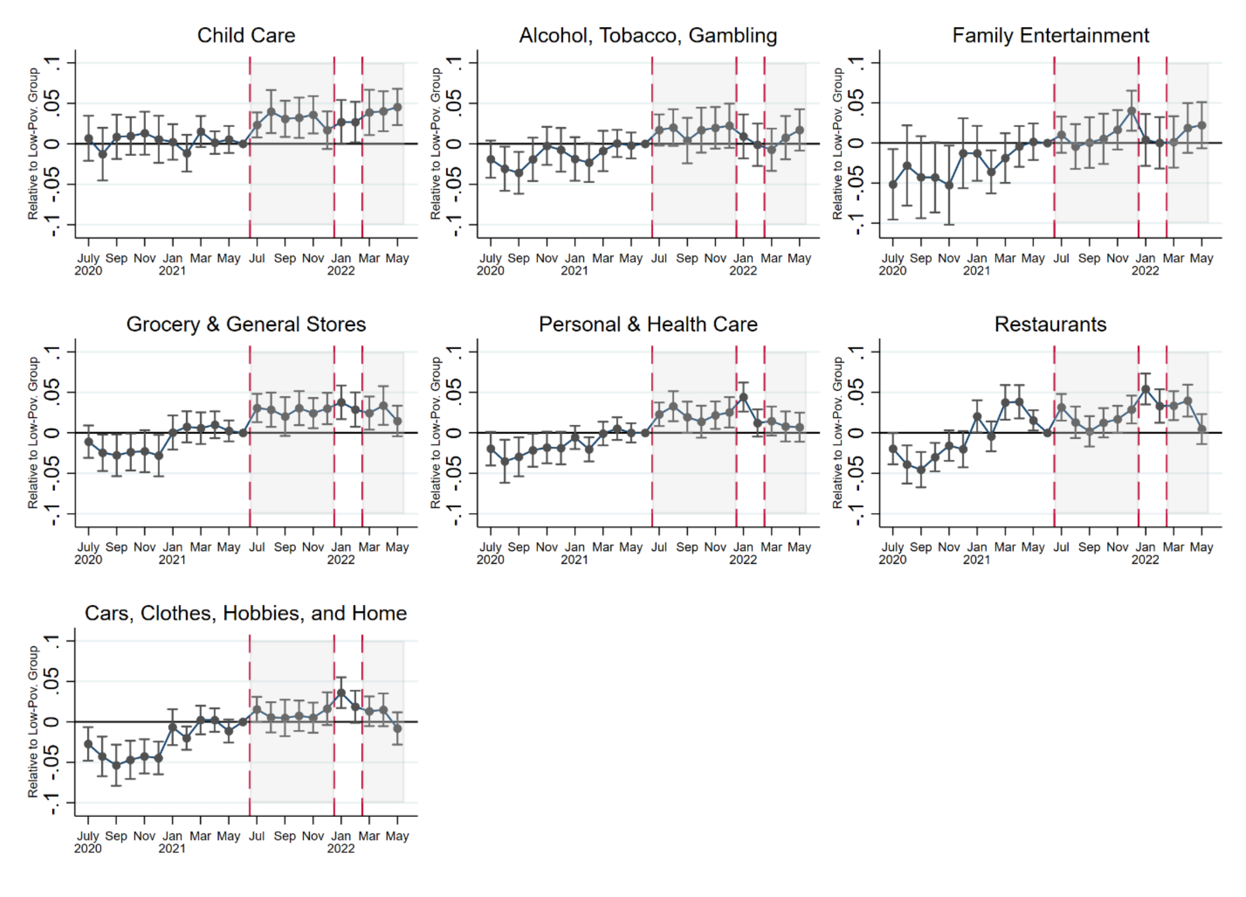

Note: The graphs display the estimated coefficients of an event-study analysis of in-person visits to different establishment types over time, comparing counties in the high and mid-poverty terciles to counties in the lowest-poverty terciles. Estimates are relative to June 2021. The gray, shaded areas represent the monthly and lump-sum CTC treatment periods, respectively. The capped horizontal bars represent 95 percent confidence intervals. The sample sizes are 70,725 (Restaurants), 68,976 (Personal & Health Care), 70,885 (General & Grocery Stores), 56,966 (Family Entertainment), 63,089 (Alcohol, Tobacco, and Gambling), 64,740 (Child Care), and 69,206 (Cars, Clothes, Hobbies, and Home).

Figure F3: Contiguous counties with common poverty status: Effects of the monthly CTC payments on seasonally-adjusted visits to different establishment types in high- and medium-poverty counties relative to low-poverty counties by CTC payment type

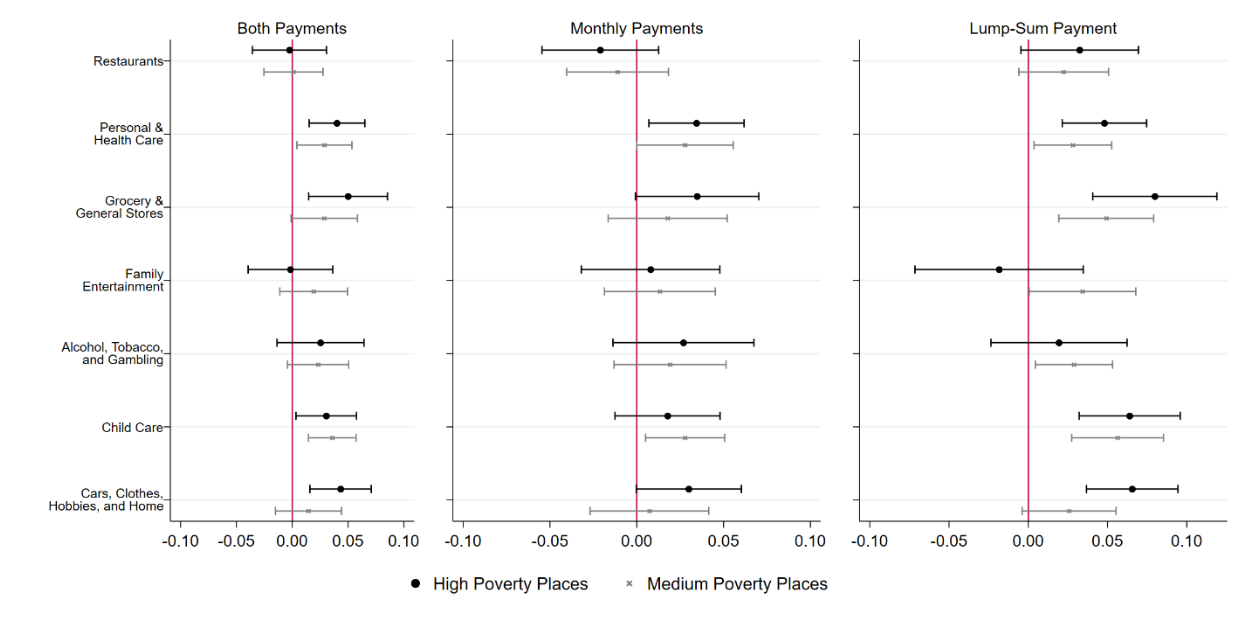

Note: Sample is restricted to counties that are in the same poverty tercile of the majority of their neighboring (contiguous) counties (64% of all counties in sample). Estimates of coefficient β_4 from model (1). See Table 1 for a description of establishment types. The capped horizontal bars represent 95 percent confidence intervals. "Monthly Payments" refers to the effects of the monthly-distributed CTC payments between July and December 2021. "Lump-Sum Payment" refers to the effects of the single, tax-time CTC payment distributed in spring 2022. The sample sizes are 35,683 (Restaurants), 34,986 (Personal & Health Care), 35,802 (General & Grocery Stores), 27,438 (Family Entertainment), 35,020 (Alcohol, Tobacco, and Gambling), 32,946 (Child Care), and 35,020 (Cars, Clothes, Hobbies, and Home).

Figure F4: Contiguous counties with common poverty status: Effects of the monthly CTC payments on log of mean spending per transaction at different establishment types in high- and medium-poverty counties relative to low-poverty counties by CTC payment type

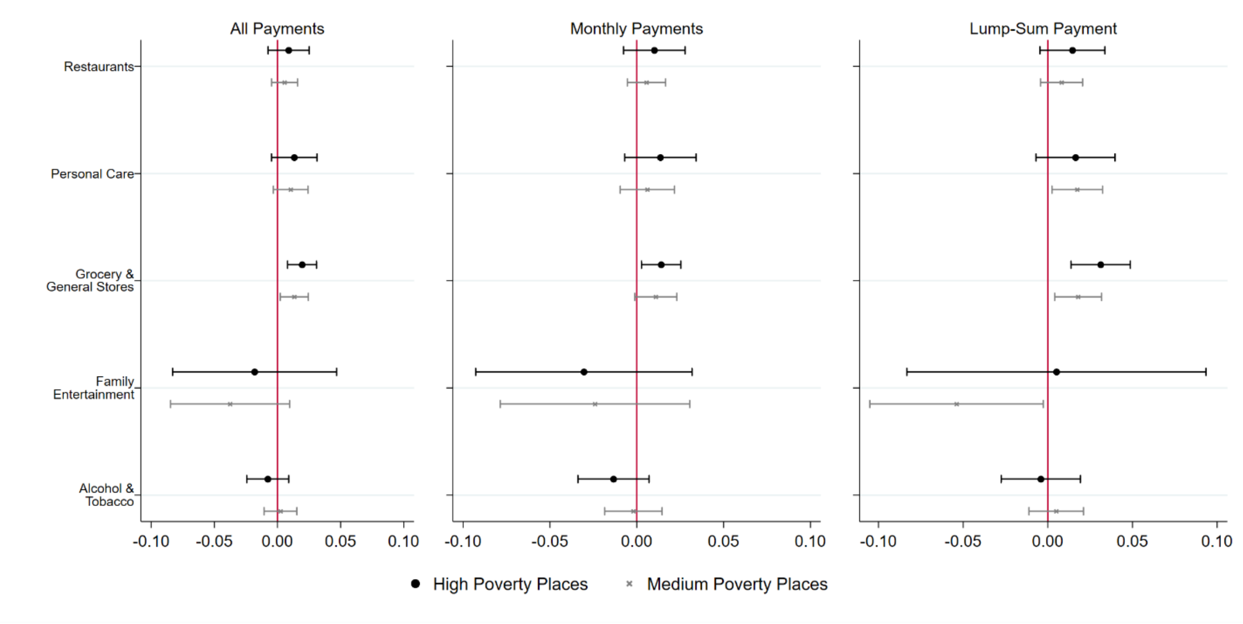

Note: Sample restricted to counties that are in the same poverty tercile of the majority of their neighboring (contiguous) counties (64% of all counties in sample). Estimates of coefficient β_4 from model (1). See Table 1 for a description of establishment types. The capped horizontal bars represent 95 percent confidence intervals. "Monthly Payments" refers to the effects of the monthly-distributed CTC payments between July and December 2021. "Lump-Sum Payment" refers to the effects of the single, tax-time CTC payment distributed in spring 2022. The sample sizes are 31,689 (Restaurants), 21,704 (Personal Care), 33,629 (General & Grocery Stores), 11,216 (Family Entertainment), and 16,006 (Alcohol and Tobacco).

APPENDIX G: Robustness Checks

Figure G1: Effects of the expanded CTC payments on seasonally-adjusted visits to establishment types in high- and medium-poverty counties relative to low-poverty counties. Monthly and lump-sum payments combined, with “partially treated” months (Jan-Feb 2022) counted as treated (left panel) and untreated (right panel)

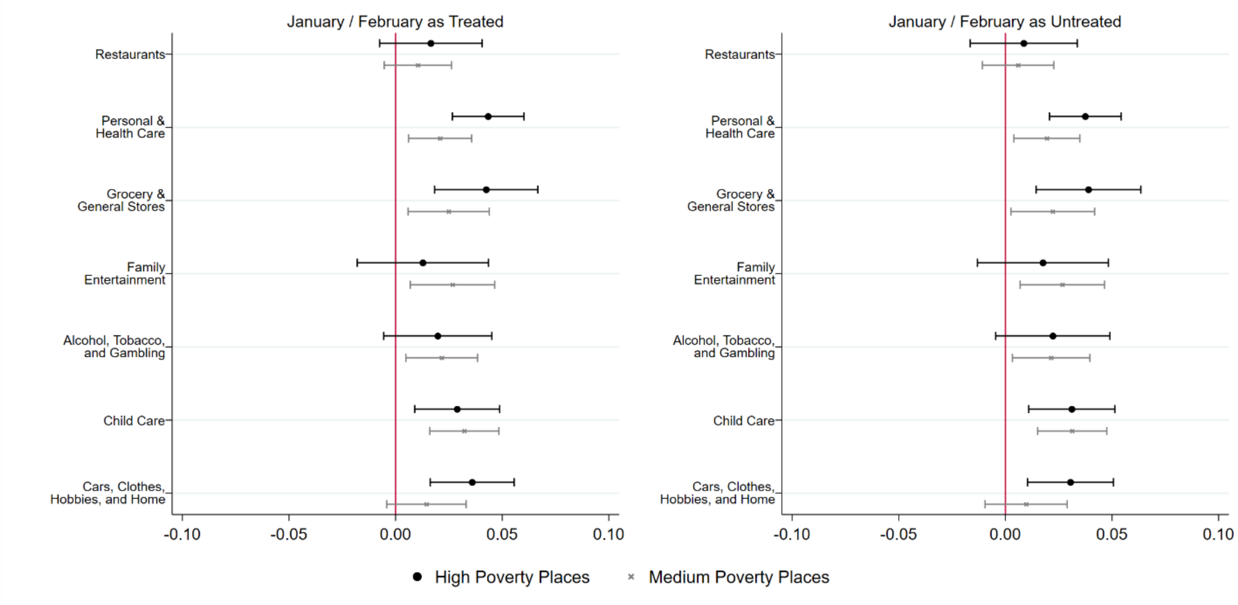

Note: Estimates of a version of model (1) in which we treat monthly payments and the lump-sum payment as a single treatment. The right panel treats “partially treated” months (Jan-Feb 2022) as untreated, the left panel as treated. The x-axis represents the estimated effect of the CTC on the percent increase in seasonally-adjusted visits relative to low-poverty counties. See Table 1 for a description of establishment types. The capped horizontal bars represent 95 percent confidence intervals. Sample sizes are 51,629 (Restaurants), 50,626 (Personal & Health Care), 51,799 (General & Grocery Stores), 40,579 (Family Entertainment), 46,155 (Alcohol, Tobacco, and Gambling), 47,464 (Child Care), and 50,643 (Cars, Clothes, Hobbies, and Home).

Figure G2: Effects of the expanded CTC payments on spending per transaction at establishment types in high- and medium-poverty counties relative to low-poverty counties. Monthly and lump-sum payments combined, with “partially treated” months (Jan-Feb 2022) counted as not treated (left panel) and treated (right panel)

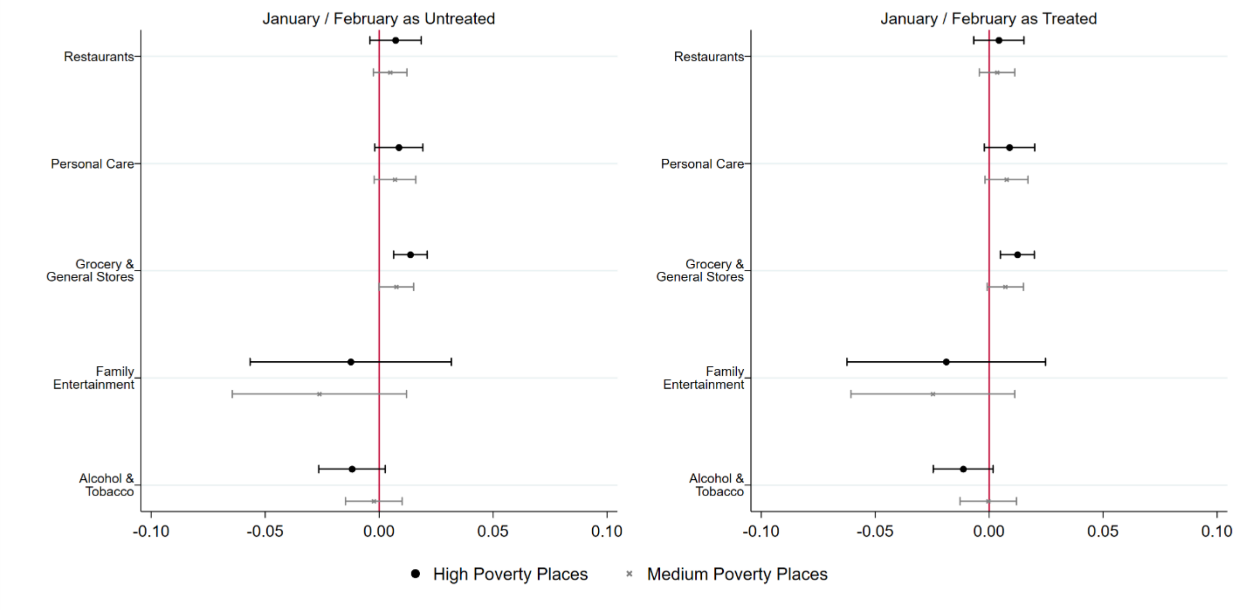

Note: Estimates of a version of model (1) in which we treat monthly payments and the lump-sum payment as a single treatment. The right panel treats “partially treated” months (Jan-Feb 2022) as untreated, the left panel as treated. The x-axis represents the estimated effect of the CTC on the percent increase in mean spending per transaction relative to low-poverty counties. See Table 1 for a description of establishment types. The capped horizontal bars represent 95 percent confidence intervals. Sample sizes are 24,928 (Restaurants), 17,606 (Personal Care), 26,438 (General & Grocery Stores), 9,463 (Family Entertainment), and 13,169 (Alcohol and Tobacco).

Figure G3: Lump-Sum Treatment Months: Includes February-May (instead of March-May): Effects of the expanded CTC payments on seasonally-adjusted visits to establishment types in high- and medium-poverty counties relative to low-poverty counties by payment type

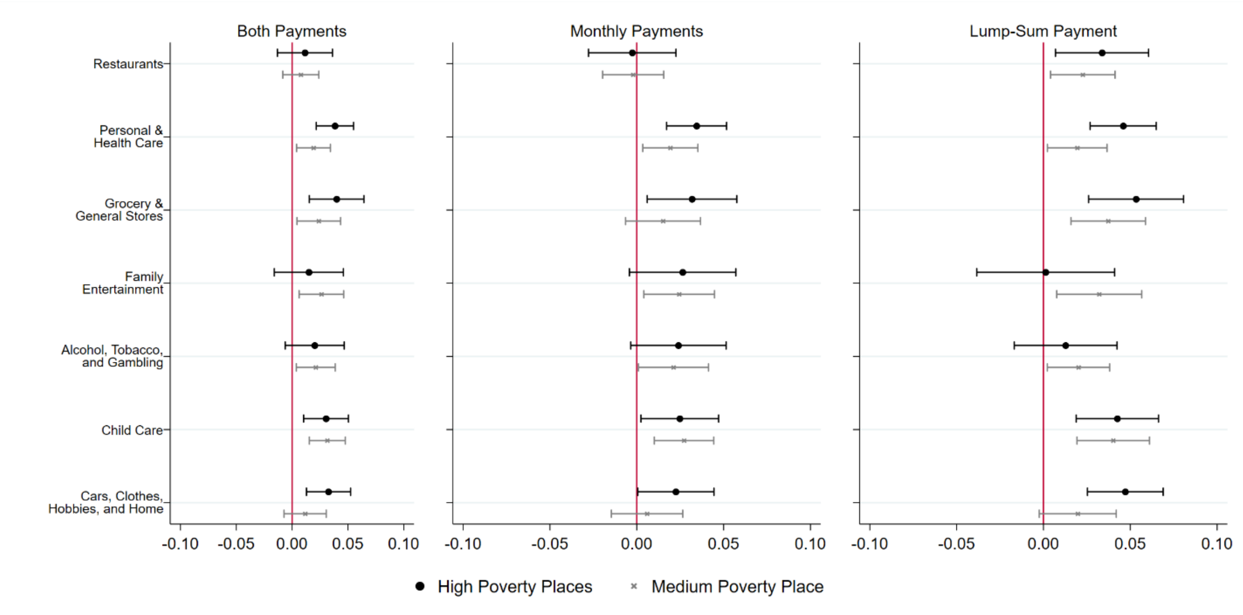

Note: Estimates of coefficient β_4 from model (1) but with lump-sum treatment months defined as February through May 2021. The x-axis represents the estimated effect of the CTC on the percent increase in mean spending per transaction relative to low-poverty counties. See Table 1 for a description of establishment types. The capped horizontal bars represent 95 percent confidence intervals. “Monthly Payments” refers to the effects of the monthly-distributed CTC payments between July and December 2021. “Lump-Sum Payment” refers to the effects of the single, tax-time CTC payment distributed in spring 2022. Sample sizes are 51,629 (Restaurants), 50,626 (Personal & Health Care), 51,799 (General & Grocery Stores), 40,579 (Family Entertainment), 46,155 (Alcohol, Tobacco, and Gambling), 47,464 (Child Care), and 50,643 (Cars, Clothes, Hobbies, and Home).

Figure G4: Lump-Sum Treatment Months: Includes February-May (instead of March-May): Effects of the expanded CTC payments on spending per transaction at establishment types in high- and medium-poverty counties relative to low-poverty counties by payment type

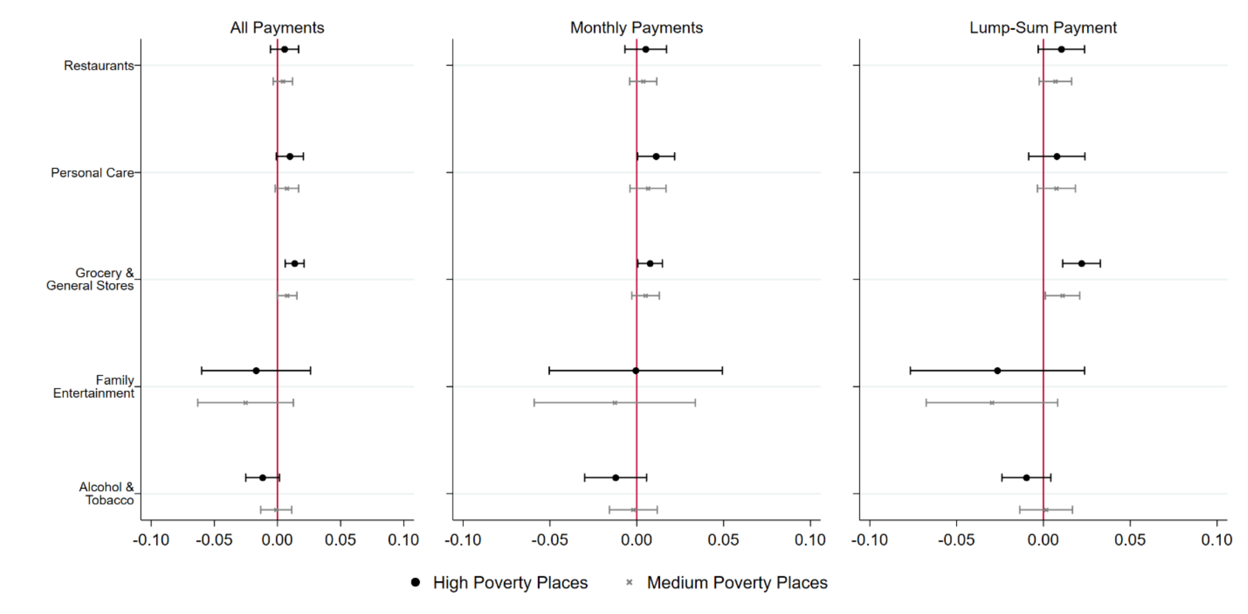

Note: Estimates of coefficient β_4 from model (1) but with lump-sum treatment months defined as February through May 2021. The x-axis represents the estimated effect of the CTC on the percent increase in mean spending per transaction relative to low-poverty counties. See Table 1 for a description of establishment types. The capped horizontal bars represent 95 percent confidence intervals. “Monthly Payments” refers to the effects of the monthly-distributed CTC payments between July and December 2021. “Lump-Sum Payment” refers to the effects of the single, tax-time CTC payment distributed in spring 2022. Sample sizes are 24,928 (Restaurants), 17,606 (Personal Care), 26,438 (General & Grocery Stores), 9,463 (Family Entertainment), and 13,169 (Alcohol and Tobacco).

Figure G5: Unweighted Estimates: Effects of the monthly CTC payments on seasonally-adjusted visits to different establishment types in high- and medium-poverty counties relative to low-poverty counties by CTC payment type

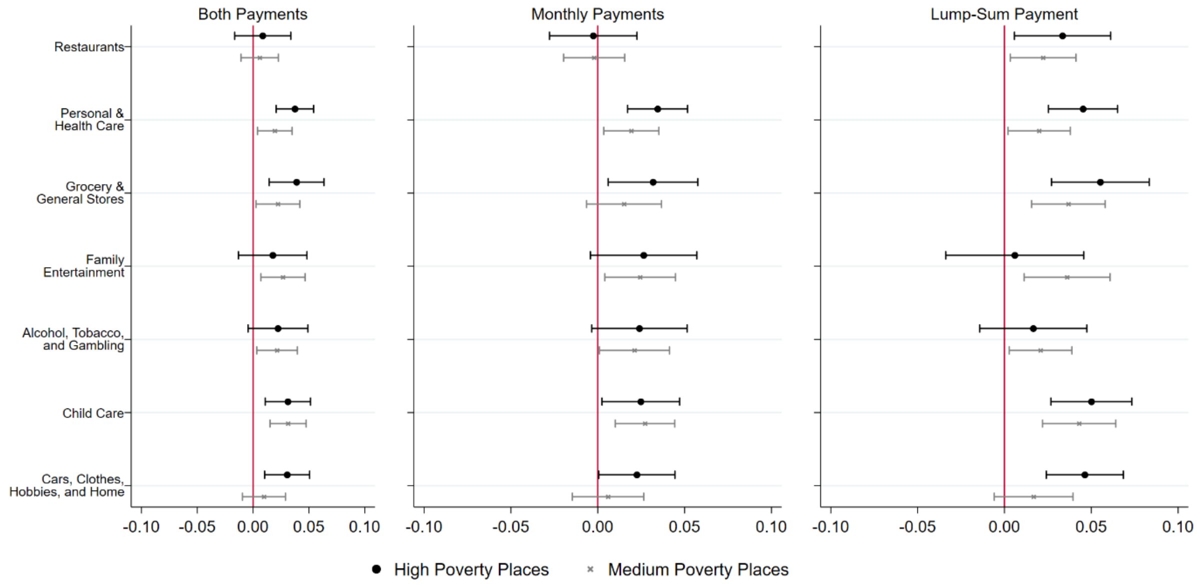

Note: Unweighted estimates of coefficient β_4 from model (1). See Table 1 for a description of establishment types. The capped horizontal bars represent 95 percent confidence intervals. "Monthly Payments" refers to the effects of the monthly-distributed CTC payments between July and December 2021. "Lump-Sum Payment" refers to the effects of the single, tax-time CTC payment distributed in spring 2022. Sample sizes are 51,629 (Restaurants), 50,626 (Personal & Health Care), 51,799 (General & Grocery Stores), 40,579 (Family Entertainment), 46,155 (Alcohol, Tobacco, and Gambling), 47,464 (Child Care), and 50,643 (Cars, Clothes, Hobbies, and Home).

Figure G6: Unweighted Estimates: Effects of the monthly CTC payments on log of mean spending per transaction at different establishment types in high- and medium-poverty counties relative to low-poverty counties by CTC payment type

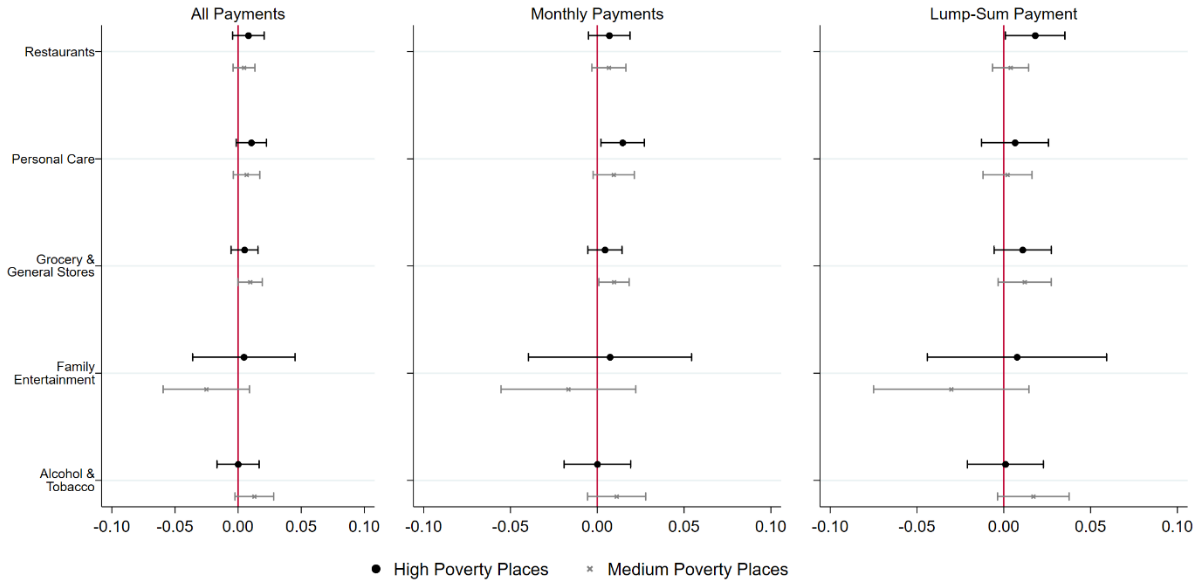

Note: Unweighted estimates of coefficient β_4 from model (1). See Table 1 for a description of establishment types. The capped horizontal bars represent 95 percent confidence intervals. "Monthly Payments" refers to the effects of the monthly-distributed CTC payments between July and December 2021. "Lump-Sum Payment" refers to the effects of the single, tax-time CTC payment distributed in spring 2022. Sample sizes are 24,928 (Restaurants), 17,606 (Personal Care), 26,438 (General & Grocery Stores), 9,463 (Family Entertainment), and 13,169 (Alcohol and Tobacco).

Figure G7: Effects of the expanded CTC payments on seasonally-adjusted visits to different establishment types, excluding counties with one or more establishment type missing

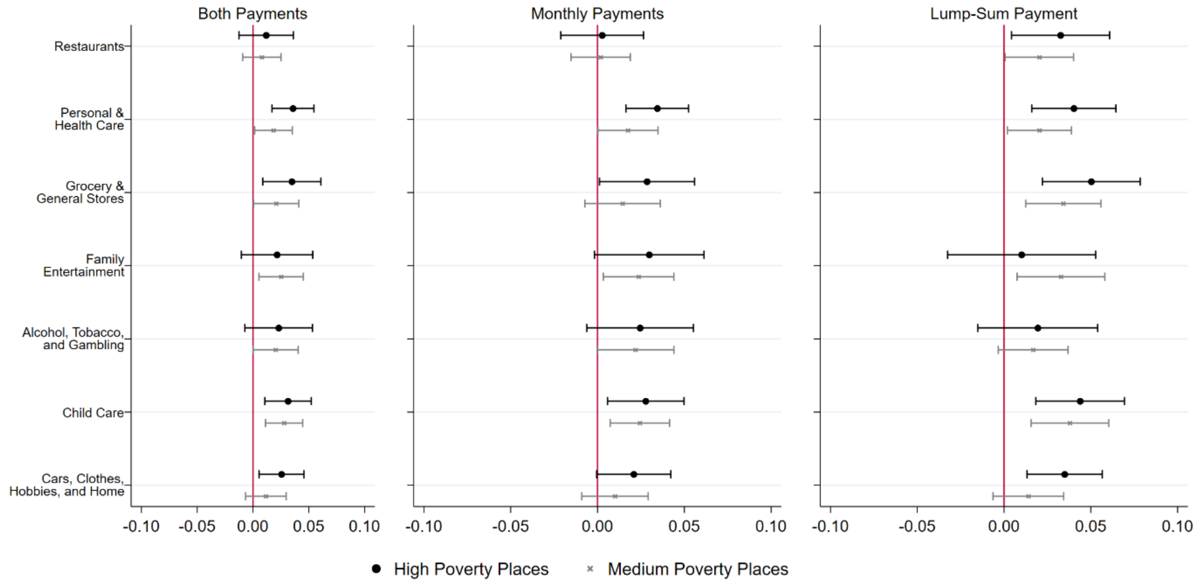

Note: Estimates of coefficient β_4 from model (1). See Table 1 for a description of establishment types. The capped horizontal bars represent 95 percent confidence intervals. “Monthly Payments” refers to the effects of the monthly-distributed CTC payments between July and December 2021. “Lump-Sum Payment” refers to the effects of the single, tax-time CTC payment distributed in spring 2022. Details on excluded counties are in Table G8. Sample sizes are 36,244 for all categories.

Figure G8: Effects of the expanded CTC payments on spending per transaction at different establishment types, excluding counties with one or more establishment type missing

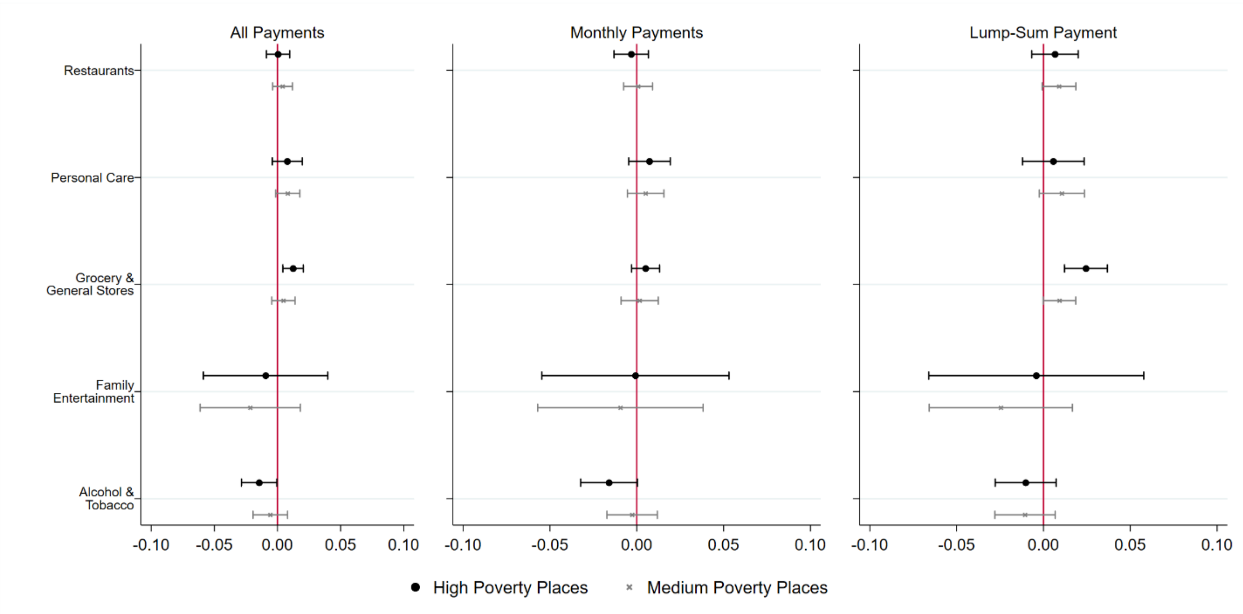

Note: Estimates of coefficient β_4 from model (1). See Table 1 for a description of establishment types. The capped horizontal bars represent 95 percent confidence intervals. "Monthly Payments" refers to the effects of the monthly-distributed CTC payments between July and December 2021. "Lump-Sum Payment" refers to the effects of the single, tax-time CTC payment distributed in spring 2022. Details on excluded counties are in Table G8. Sample sizes are 14,655 for all categories.

Figure G9: Estimated effect of the CTC on in-person visits to children's and family clothing stores relative to other clothing stores by poverty status and payment type

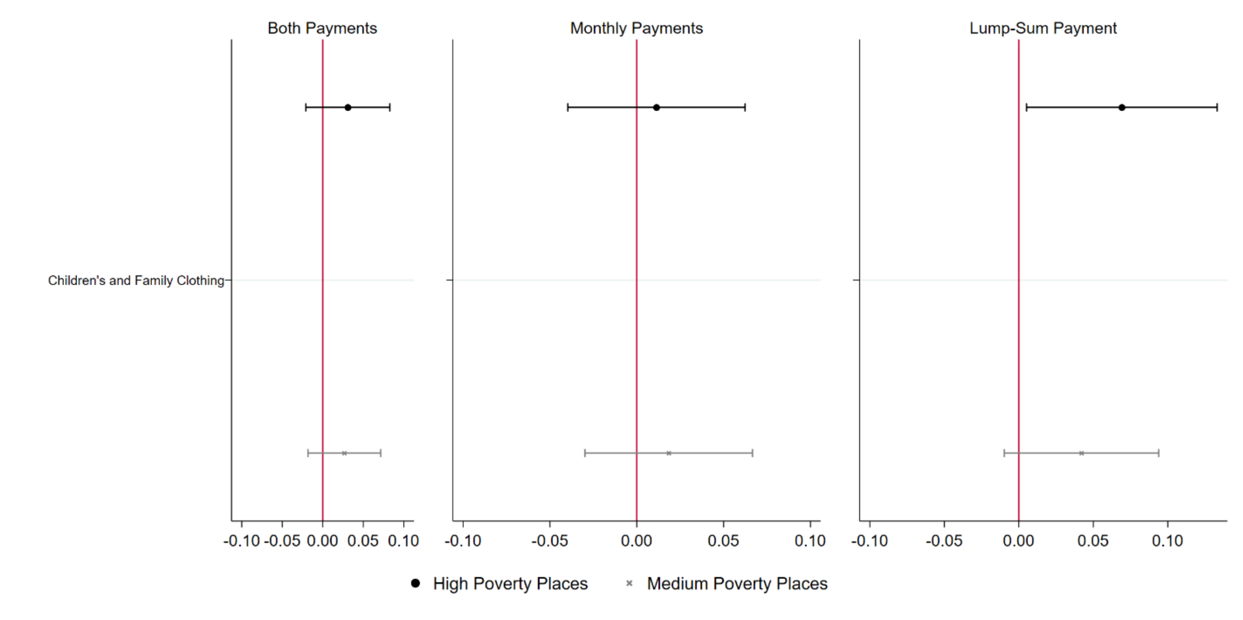

Note: The figure presents the estimated coefficients on the triple interaction of the poverty bin of the county (reference: low-poverty counties), the post-CTC treatment indicator, and spending on children's and family clothing (reference: non-children's and non-family clothing establishments) in an augmented version of model (1) in which we also difference with respect to clothing type. Sample size = 129,812. The capped horizontal bars represent 95 percent confidence intervals.

Figure G10: Event study specification for visits to child care centers by selection of fixed effects

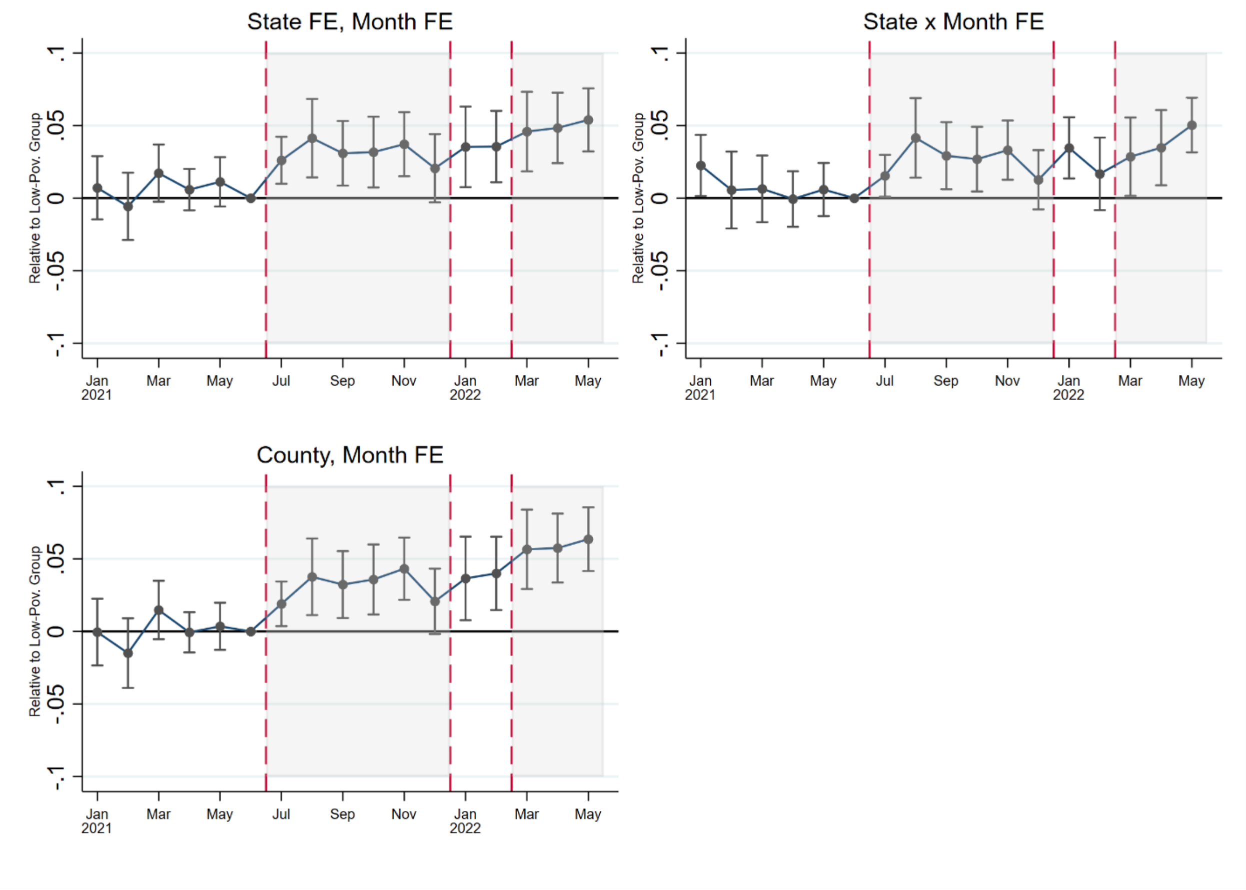

Note: The graphs display the estimated coefficients of an event-study analysis of visits to child care centers over time, comparing counties in the high and mid-poverty terciles to counties in the lowest-poverty tercile and using different regression specifications. Estimates are relative to June 2021. The gray, shaded areas represent the monthly and lump-sum CTC treatment periods, respectively. Sample size = 47,464. The capped horizontal bars represent 95 percent confidence intervals.

Figure G11: Event study specification for visits to grocery and general stores by selection of fixed effects

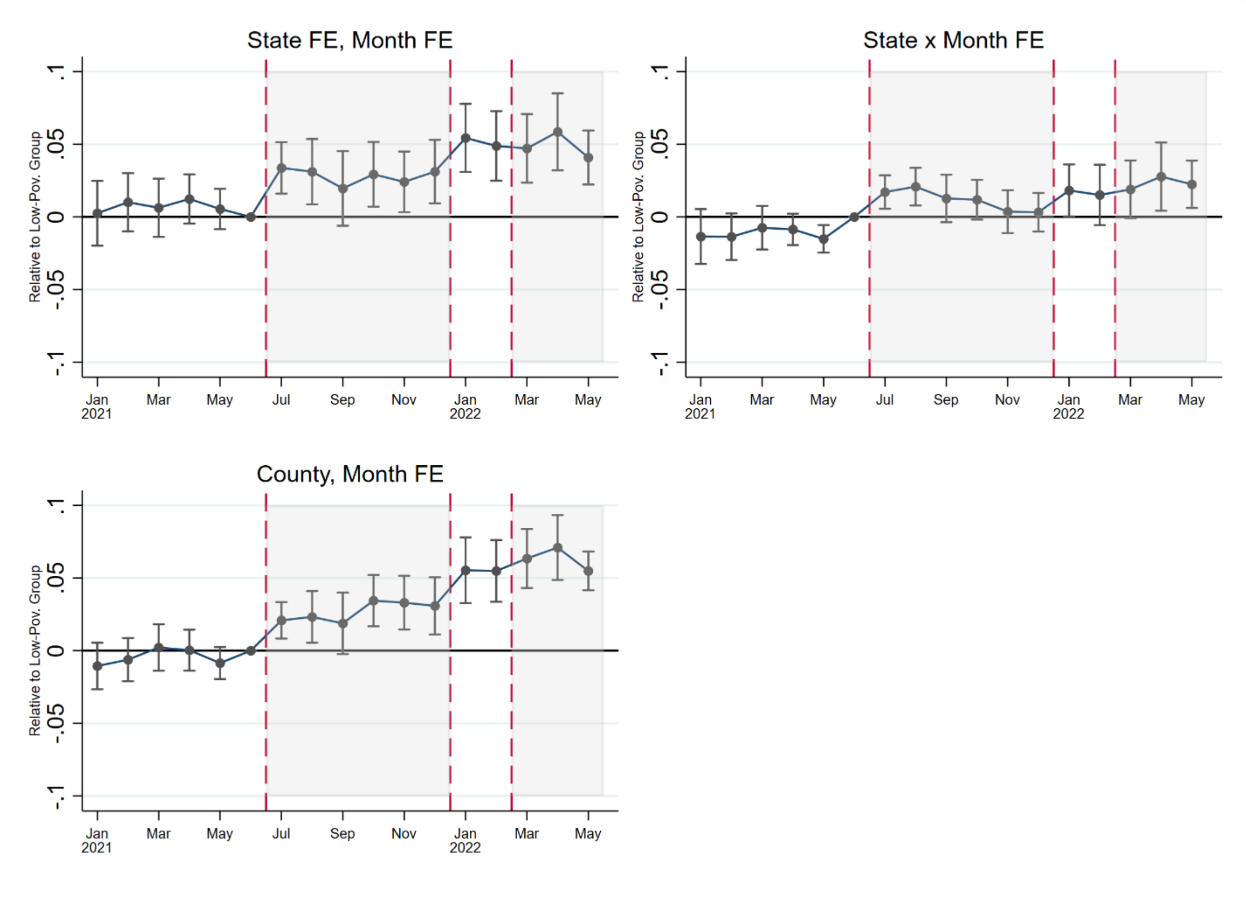

Note: The graphs display the estimated coefficients of an event-study analysis of visits to grocery and general stores over time, comparing counties in the high and mid-poverty terciles to counties in the lowest-poverty tercile and using different regression specifications. Estimates are relative to June 2021. The gray, shaded areas represent the monthly and lump-sum CTC treatment periods, respectively. Sample size = 51,799. The capped horizontal bars represent 95 percent confidence intervals.

Figure G12: Event study specification for visits to personal and health care establishments by selection of fixed effects

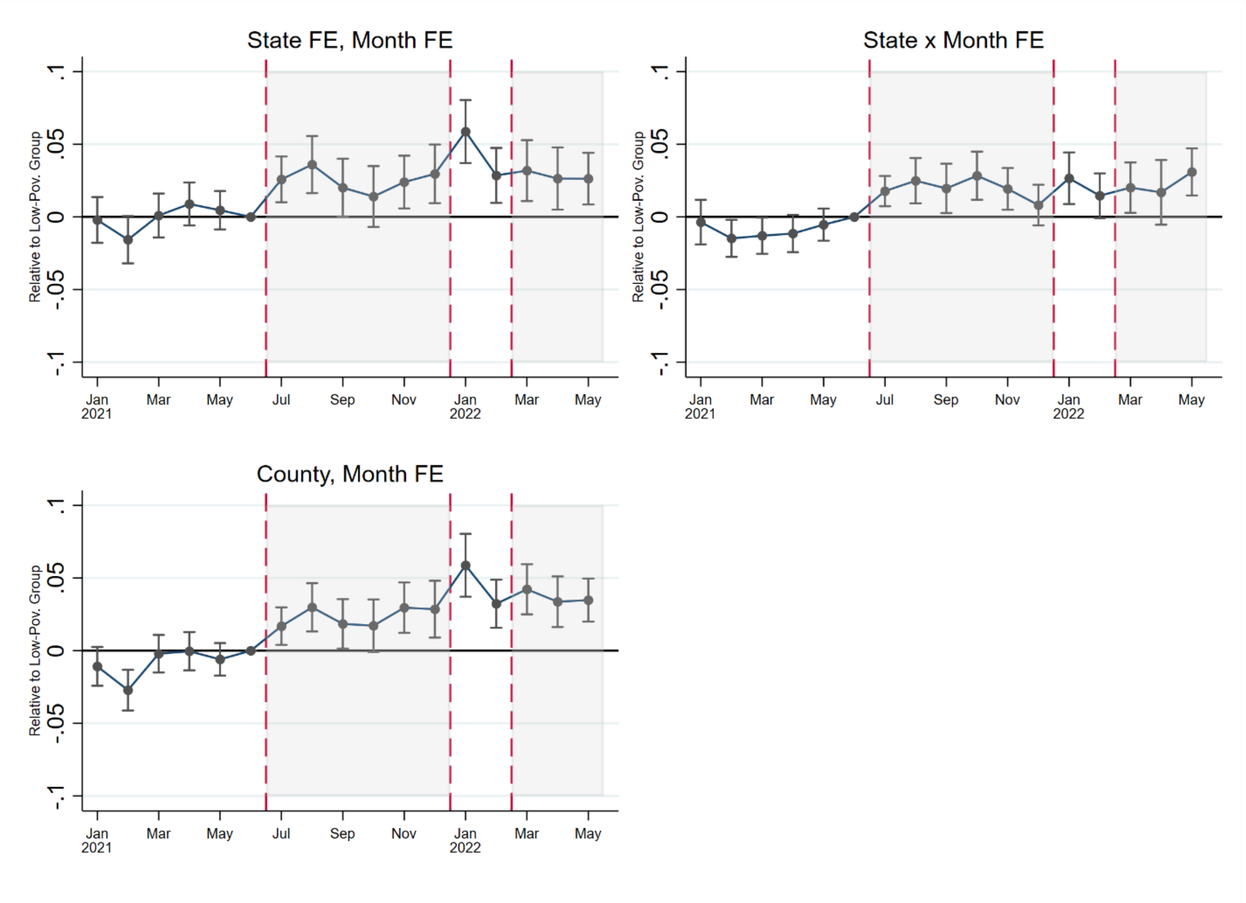

Note: The graphs display the estimated coefficients of an event-study analysis of visits to personal and health care establishments over time, comparing counties in the high and mid-poverty terciles to counties in the lowest-poverty tercile and using different regression specifications. Estimates are relative to June 2021. The gray, shaded areas represent the monthly and lump-sum CTC treatment periods, respectively. Sample size = 50,626. The capped horizontal bars represent 95 percent confidence intervals.

Figure G13: Event study specification for spending at grocery and general stores by selection of fixed effects

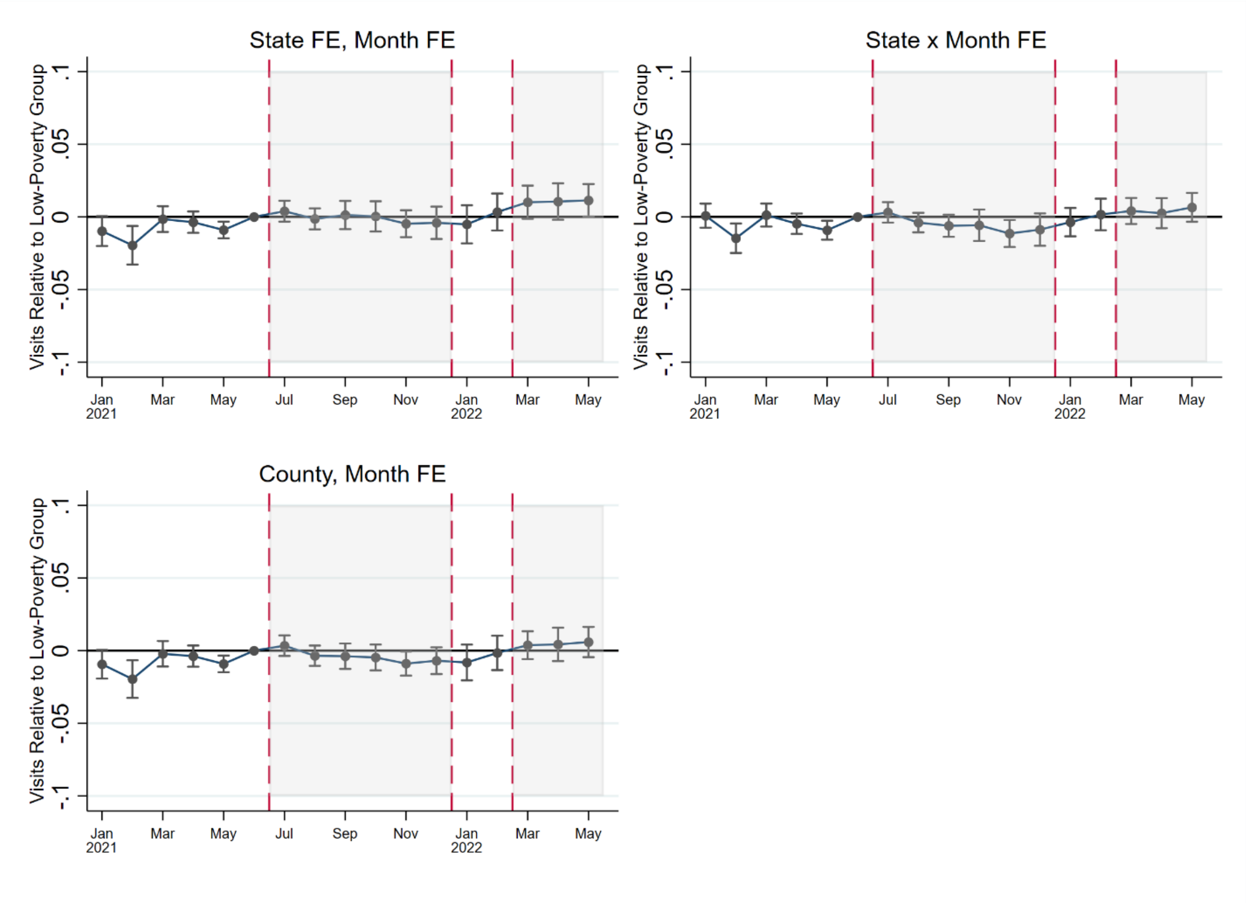

Note: The graphs display the estimated coefficients of an event-study analysis of spending per transaction at grocery and general stores, comparing counties in the high and mid-poverty terciles to counties in the lowest-poverty tercile and using different regression specifications. Estimates are relative to June 2021. The gray, shaded areas represent the monthly and lump-sum CTC treatment periods, respectively. Sample size = 26,438. The capped horizontal bars represent 95 percent confidence intervals.

Table G1: Regression Tables: Mobility Estimates by Treatment Type

**Both Treatments**	(1)<br>Alcohol, Tobacco, and Gambling	(2)<br>Restaurants	(3)<br>Family Entertainment	(4)<br>Personal and Health Care
Medium Poverty X Post-CTC Expansion	0.021*<br>(0.009)<br>[P=0.02]	0.006<br>(0.008)<br>[P=0.48]	0.027**<br>(0.010)<br>[P=0.01]	0.019*<br>(0.008)<br>[P=0.01]
High Poverty X Post-CTC Expansion	0.022<br>(0.013)<br>[P=0.10]	0.009<br>(0.013)<br>[P=0.49]	0.018<br>(0.015)<br>[P=0.25]	0.037***<br>(0.008)<br>[P=0.00]

	(5)<br>Grocery and General Stores	(6)<br>Cars, Clothes, Hobbies, and Home	(7)<br>Child Care
Medium Poverty X Post-CTC Expansion	0.022*<br>(0.010)<br>[P=0.03]	0.010<br>(0.010)<br>[P=0.31]	0.031***<br>(0.008)<br>[P=0.00]
High Poverty X Post-CTC Expansion	0.039**<br>(0.012)<br>[P=0.00]	0.031**<br>(0.010)<br>[P=0.00]	0.031**<br>(0.010)<br>[P=0.00]

**Monthly Treatment**	(1)<br>Alcohol, Tobacco, and Gambling	(2)<br>Restaurants	(3)<br>Family Entertainment	(4)<br>Personal and Health Care
Medium Poverty X Post-CTC Expansion	0.021*<br>(0.010)<br>[P=0.04]	-0.002<br>(0.009)<br>[P=0.81]	0.024*<br>(0.010)<br>[P=0.02]	0.019*<br>(0.008)<br>[P=0.02]
High Poverty X Post-CTC Expansion	0.024<br>(0.014)<br>[P=0.09]	-0.003<br>(0.013)<br>[P=0.84]	0.026<br>(0.015)<br>[P=0.00]	0.034***<br>(0.009)<br>[P=0.00]

	(5)<br>Grocery and General Stores	(6)<br>Cars, Clothes, Hobbies, and Home	(7)<br>Child Care
Medium Poverty X Post-CTC Expansion	0.015<br>(0.011)<br>[P=0.17]	0.006<br>(0.010)<br>[P=0.57]	0.027**<br>(0.009)<br>[P=0.00]
High Poverty X Post-CTC Expansion	0.032*<br>(0.013)<br>[P=0.02]	0.022*<br>(0.011)<br>[P=0.04]	0.025*<br>(0.011)<br>[P=0.03]

**Lump-Sum Treatments**	(1) Alcohol, Tobacco, and Gambling	(2) Restaurants	(3) Family Entertainment	(4) Personal and Health Care
Medium Poverty X Post-CTC Expansion	0.021* (0.009) [P=0.03]	0.022* (0.009) [P=0.02]	0.036** (0.012) [P=0.01]	0.020* (0.009) [P=0.03]
High Poverty X Post-CTC Expansion	0.017 (0.015) [P=0.29]	0.033* (0.014) [P=0.02]	0.006 (0.020) [P=0.77]	0.045*** (0.010) [P=0.00]
	(5) Grocery and General Stores	(6) Cars, Clothes, Hobbies, and Home	(7) Child Care	
Medium Poverty X Post-CTC Expansion	0.037*** (0.011) [P=0.00]	0.017 (0.011) [P=0.14]	0.043*** (0.011) [P=0.00]	
High Poverty X Post-CTC Expansion	0.055*** (0.014) [P=0.00]	0.046*** (0.011) [P=0.00]	0.050*** (0.012) [P=0.00]	

Note: Estimates of coefficient β_4 from model (1). The results are presented in Figure 1. Coefficient estimates for the extended set of controls in columns (4), (5) and (7) are reported in Table G3. Standard errors in parentheses. * $p < 0.05$, ** $p < 0.01$, *** $p < 0.001$. All P values are based on two-tailed tests. No adjustments were made for multiple comparisons.

Table G2: Regression Tables: Spending Estimates by Treatment Type

**Both Treatments**	(1)<br>Alcohol and Tobacco	(2)<br>Restaurants	(3)<br>Personal Care	(4)<br>Family Entertainment	(5)<br>Grocery and General Stores
Medium Poverty X Post-CTC Expansion	-0.002<br>(0.006)<br>[P=0.71]	0.005<br>(0.004)<br>[P=0.19]	0.007<br>(0.005)<br>[P=0.14]	-0.026<br>(0.019)<br>[P=0.17]	0.008<br>(0.004)<br>[P=0.05]
High Poverty X Post-CTC Expansion	-0.012<br>(0.007)<br>[P=0.11]	0.007<br>(0.006)<br>[P=0.21]	0.009<br>(0.005)<br>[P=0.11]	-0.012<br>(0.022)<br>[P=0.57]	0.014***<br>(0.004)<br>[P=0.00]

**Monthly Treatment**	(1)<br>Alcohol and Tobacco	(2)<br>Restaurants	(3)<br>Personal Care	(4)<br>Family Entertainment	(5)<br>Grocery and General Stores
Medium Poverty X Post-CTC Expansion	-0.002<br>(0.007)<br>[P=0.77]	0.004<br>(0.004)<br>[P=0.35]	0.006<br>(0.005)<br>[P=0.22]	-0.013<br>(0.023)<br>[P=0.59]	0.005<br>(0.004)<br>[P=0.20]
High Poverty X Post-CTC Expansion	-0.012<br>(0.009)<br>[P=0.18]	0.005<br>(0.006)<br>[P=0.40]	0.011*<br>(0.005)<br>[P=0.04]	-0.001<br>(0.025)<br>[P=0.98]	0.008*<br>(0.004)<br>[P=0.03]

**Lump-Sum Treatment**	(1)<br>Alcohol and Tobacco	(2)<br>Restaurants	(3)<br>Personal Care	(4)<br>Family Entertainment	(5)<br>Grocery and General Stores
Medium Poverty X Post-CTC Expansion	-0.002<br>(0.008)<br>[P=0.80]	0.009<br>(0.004)<br>[P=0.05]	0.006<br>(0.005)<br>[P=0.27]	-0.033<br>(0.020)<br>[P=0.11]	0.012*<br>(0.005)<br>[P=0.02]
High Poverty X Post-CTC Expansion	-0.010<br>(0.008)<br>[P=0.20]	0.016*<br>(0.007)<br>[P=0.03]	0.004<br>(0.008)<br>[P=0.61]	-0.016<br>(0.027)<br>[P=0.56]	0.025***<br>(0.006)<br>[P=0.00]

Note: Estimates of coefficient β_4 from model (1). Results are presented in Figure 3. Coefficient estimates for the extended set of controls in column (5) are reported in Table G3. Standard errors in parentheses. * p < 0.05, ** p < 0.01, *** p < 0.001. All P values are based on two-tailed tests. No adjustments were made for multiple comparisons.

Table G3: Child Care, Mobility Estimates Across Alternative Model Specifications

	Child Care: Pre-Trend	Child Care: All Payments	Child Care: Monthly Payments	Child Care: Lump-Sum Payments
State FE, Month FE				
Medium Poverty X Treatment	-0.004	0.031***	0.027**	0.043***
	(0.002)	(0.008)	(0.009)	(0.011)
	[P=0.11]	[P=0.00]	[P=0.00]	[P=0.00]
High Poverty X Treatment	0.006	0.031**	0.025*	0.050***
	(0.003)	(0.010)	(0.011)	(0.012)
	[P=0.09]	[P=0.00]	[P=0.03]	[P=0.00]
State x Month FE				
Medium Poverty X Treatment	-0.006*	0.023**	0.021*	0.030**
	(0.002)	(0.008)	(0.008)	(0.010)
	[P=0.03]	[P=0.00]	[P=0.01]	[P=0.01]
High Poverty X Treatment	0.002	0.025*	0.022*	0.034**
	(0.003)	(0.010)	(0.011)	(0.011)
	[P=0.65]	[P=0.01]	[P=0.04]	[P=0.00]
County FE, Month FE				
Medium Poverty X Treatment	-0.003	0.038***	0.035***	0.052***
	(0.002)	(0.008)	(0.008)	(0.010)
	[P=0.24]	[P=0.00]	[P=0.00]	[P=0.00]
High Poverty X Treatment	0.008*	0.043***	0.038**	0.069***
	(0.003)	(0.010)	(0.012)	(0.011)
	[P=0.02]	[P=0.00]	[P=0.00]	[P=0.00]
Within-State Poverty Bins (State, Month FE)				
Medium Poverty X Treatment	0.002	0.020**	0.020*	0.023*
	(0.003)	(0.007)	(0.008)	(0.009)
	[P=0.58]	[P=0.01]	[P=0.01]	[P=0.01]
High Poverty X Treatment	0.001	0.022**	0.022*	0.026**
	(0.002)	(0.008)	(0.009)	(0.009)
	[P=0.60]	[P=0.01]	[P=0.01]	[P=0.01]

Note: Standard errors in parentheses. * $p < 0.05$, ** $p < 0.01$, *** $p < 0.001$. All P values are based on two-tailed tests. No adjustments were made for multiple comparisons.

Table G4: Personal and Health Care, Mobility Estimates Across Alternative Model Specifications

	Personal and Health Care: Pre-Trend	Personal and Health Care: All Payments	Personal and Health Care: Monthly Payments	Personal and Health Care: Lump-Sum Payments
State FE, Month FE				
Medium Poverty X Treatment	0.002<br>(0.001)<br>[P=0.16]	0.019*<br>(0.008)<br>[P=0.01]	0.019*<br>(0.008)<br>[P=0.02]	0.020*<br>(0.009)<br>[P=0.03]
High Poverty X Treatment	0.003<br>(0.002)<br>[P=0.16]	0.037***<br>(0.008)<br>[P=0.00]	0.034***<br>(0.009)<br>[P=0.00]	0.045***<br>(0.010)<br>[P=0.00]
State x Month FE				
Medium Poverty X Treatment	0.001<br>(0.001)<br>[P=0.26]	0.020***<br>(0.005)<br>[P=0.00]	0.021***<br>(0.005)<br>[P=0.00]	0.022**<br>(0.007)<br>[P=0.01]
High Poverty X Treatment	0.001<br>(0.001)<br>[P=0.34]	0.046***<br>(0.006)<br>[P=0.00]	0.045***<br>(0.006)<br>[P=0.00]	0.051***<br>(0.009)<br>[P=0.00]
County FE, Month FE				
Medium Poverty X Treatment	0.003<br>(0.001)<br>[P=0.06]	0.026***<br>(0.007)<br>[P=0.00]	0.023***<br>(0.006)<br>[P=0.00]	0.029***<br>(0.007)<br>[P=0.00]
High Poverty X Treatment	0.005**<br>(0.002)<br>[P=0.01]	0.051***<br>(0.008)<br>[P=0.00]	0.044***<br>(0.008)<br>[P=0.00]	0.067***<br>(0.008)<br>[P=0.00]
Within-State Poverty Bins (State, Month FE)				
Medium Poverty X Treatment	0.002<br>(0.003)<br>[P=0.58]	0.020**<br>(0.007)<br>[P=0.01]	0.020*<br>(0.008)<br>[P=0.01]	0.023*<br>(0.009)<br>[P=0.01]
High Poverty X Treatment	0.001<br>(0.002)<br>[P=0.60]	0.022**<br>(0.008)<br>[P=0.01]	0.022*<br>(0.009)<br>[P=0.01]	0.026**<br>(0.009)<br>[P=0.01]

Note: Standard errors in parentheses. * $p < 0.05$, ** $p < 0.01$, *** $p < 0.001$. All P values are based on two-tailed tests. No adjustments were made for multiple comparisons.

Table G5: Grocery and General Stores, Mobility Estimates Across Alternative Model Specifications

	Grocery and General Stores: Pre-Trend	Grocery and General Stores: All Payments	Grocery and General Stores: Monthly Payments	Grocery and General Stores: Lump-Sum Payments
State FE, Month FE				
Medium Poverty X Treatment	-0.001	0.022*	0.015	0.037***
	(0.002)	(0.010)	(0.011)	(0.011)
	[P=0.79]	[P=0.03]	[P=0.17]	[P=0.00]
High Poverty X Treatment	0.000	0.039**	0.032*	0.055***
	(0.002)	(0.012)	(0.013)	(0.014)
	[P=0.98]	[P=0.00]	[P=0.02]	[P=0.00]
State x Month FE				
Medium Poverty X Treatment	0.001	0.019**	0.016*	0.028**
	(0.002)	(0.007)	(0.006)	(0.009)
	[P=0.52]	[P=0.01]	[P=0.02]	[P=0.00]
High Poverty X Treatment	0.004*	0.037***	0.037***	0.045***
	(0.002)	(0.009)	(0.008)	(0.012)
	[P=0.04]	[P=0.00]	[P=0.00]	[P=0.00]
County FE, Month FE				
Medium Poverty X Treatment	0.001	0.032***	0.021*	0.052***
	(0.001)	(0.008)	(0.009)	(0.009)
	[P=0.54]	[P=0.00]	[P=0.03]	[P=0.00]
High Poverty X Treatment	0.004*	0.058***	0.044***	0.090***
	(0.002)	(0.010)	(0.011)	(0.011)
	[P=0.01]	[P=0.00]	[P=0.00]	[P=0.00]
Within-County Poverty Bins (State, Month FE)				
Medium Poverty X Treatment	0.003	0.021***	0.017**	0.030***
	(0.001)	(0.005)	(0.005)	(0.006)
	[P=0.06]	[P=0.00]	[P=0.00]	[P=0.00]
High Poverty X Treatment	0.004*	0.042***	0.042***	0.049***
	(0.002)	(0.007)	(0.007)	(0.008)
	[P=0.01]	[P=0.00]	[P=0.00]	[P=0.00]

Note: Standard errors in parentheses. * $p < 0.05$, ** $p < 0.01$, *** $p < 0.001$. All P values are based on two-tailed tests. No adjustments were made for multiple comparisons.

Table G6: Grocery and General Stores, Spending Estimates Across Alternative Model Specifications

	Grocery and General Stores: Pre-Trend	Grocery and General Stores: All Payments	Grocery and General Stores: Monthly Payments	Grocery and General Stores: Lump-Sum Payments
State FE, Month FE				
Medium Poverty X Treatment	0.002	0.008	0.005	0.012*
	(0.001)	(0.004)	(0.004)	(0.005)
	[P=0.08]	[P=0.05]	[P=0.20]	[P=0.02]
High Poverty X Treatment	0.003	0.014***	0.008*	0.025***
	(0.002)	(0.004)	(0.004)	(0.006)
	[P=0.07]	[P=0.00]	[P=0.03]	[P=0.00]
State x Month FE				
Medium Poverty X Treatment	0.000	0.002	-0.001	0.007
	(0.001)	(0.003)	(0.003)	(0.004)
	[P=0.72]	[P=0.49]	[P=0.76]	[P=0.12]
High Poverty X Treatment	-0.000	0.003	-0.004	0.016**
	(0.001)	(0.003)	(0.003)	(0.006)
	[P=0.75]	[P=0.35]	[P=0.24]	[P=0.01]
County FE, Month FE				
Medium Poverty X Treatment	0.002	0.005	0.002	0.008
	(0.001)	(0.003)	(0.003)	(0.004)
	[P=0.07]	[P=0.14]	[P=0.65]	[P=0.06]
High Poverty X Treatment	0.003*	0.009*	0.001	0.019**
	(0.002)	(0.004)	(0.003)	(0.006)
	[P=0.03]	[P=0.02]	[P=0.79]	[P=0.00]
Within-County Poverty Bins (State, Month FE)				
Medium Poverty X Treatment	0.000	-0.001	-0.004	0.005
	(0.001)	(0.003)	(0.003)	(0.004)
	[P=0.89]	[P=0.76]	[P=0.18]	[P=0.25]
High Poverty X Treatment	-0.001	0.003	-0.002	0.013*
	(0.001)	(0.004)	(0.004)	(0.005)
	[P=0.45]	[P=0.35]	[P=0.55]	[P=0.01]

Note: Standard errors in parentheses. * $p < 0.05$, ** $p < 0.01$, *** $p < 0.001$. All P values are based on two-tailed tests. No adjustments were made for multiple comparisons.

Table G7: Characteristics of all counties and counties missing 1 or more establishment types

	**All Counties**	**Counties Missing 1 or More Establishment Types**
Mean Population of Census Tracts	106,462	12,796
Population Density (Log)	4.89	3.37
Poverty Rate	16.1%	18.1%
Low Poverty County	32.8%	25.7%
Medium Poverty County	32.9%	27.7%
High Poverty County	34.2%	46.6%
# Counties in Sample	3,082	950
Population Coverage	328,118,758	12,156,716
Share of U.S. Population Covered	99.6%	3.7%

Note: Our primary analyses use data from all counties, inclusive of the "counties missing 1 or more establishment types." In a secondary set of analyses presented in Figures G7 and G8, we limit our sample to counties not missing 1 or more establishment types (i.e. excluding the 950 counties in the right column).

APPENDIX H: Classification of Poverty Bins across Counties

Figure H1: County poverty classification based on national distribution of poverty rate

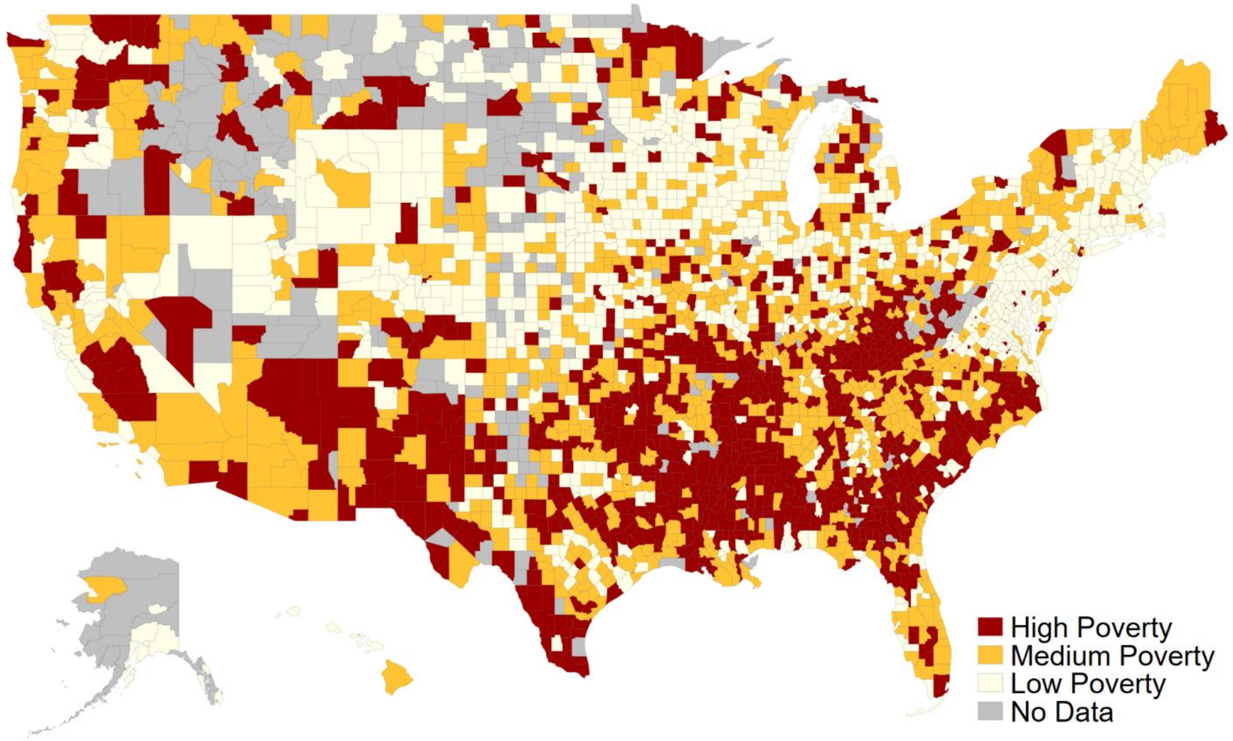

Note: This figure visualizes the counties included in the sample and their poverty status in the three-category approach applied in the primary estimates. Poverty bins are defined based on the national distribution of the poverty rate. See below for an alternative version that captures within-state variation in poverty rather than national variation. We used the maptile command provided by Michael Stepner (https://michaelstepner.com/maptile/).

Figure H2: County poverty classification based on state-level distribution of poverty rate

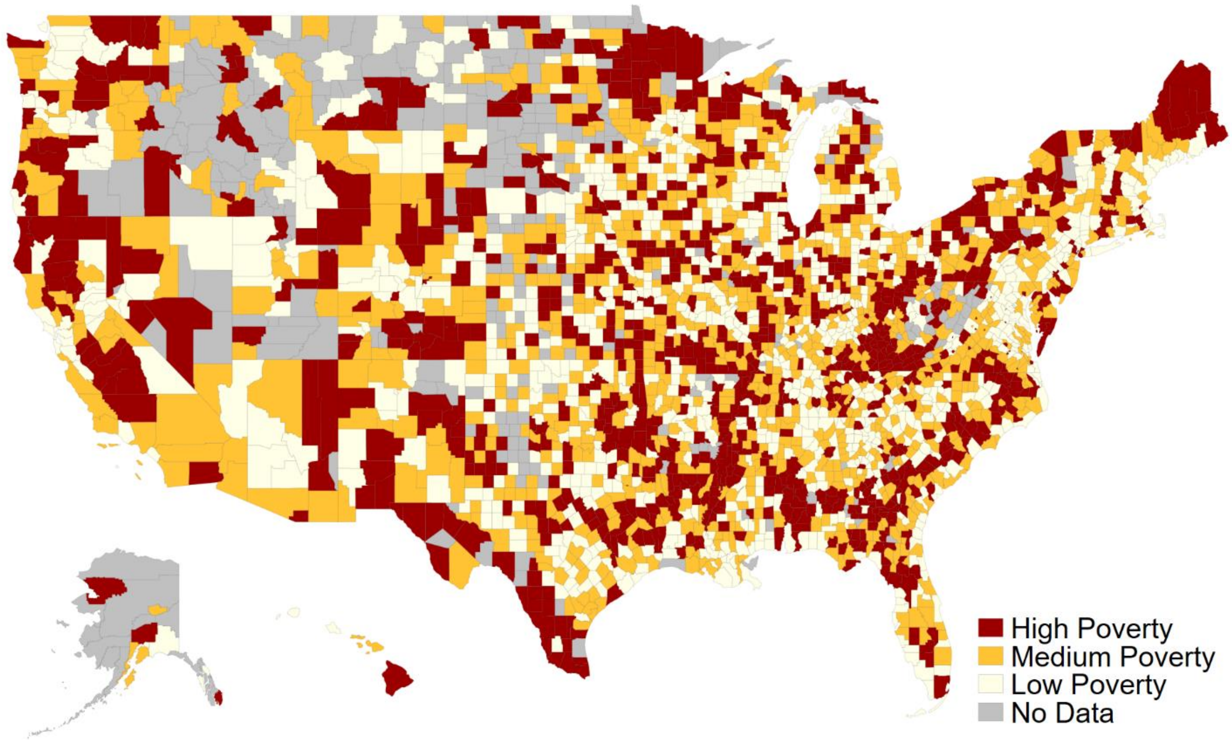

Note: This figure visualizes the counties included in the sample and their poverty status in the three-category approach applied in our within-state estimates. Poverty bins are defined based on the state-level distribution of the poverty rate. We used the maptile command provided by Michael Stepner (https://michaelstepner.com/maptile/).

Appendix I: North American Industry Classification System (NAICS) Codes

Table I1: North American Industry Classification System (NAICS) Codes for Categories of Interest

#	Category	2017 NAICS Codes
1	Automobile, Clothes, Hobbies, and Home Supplies	441110, 441120, 441210, 441222, 441228, 441310, 441320, 811111, *811112*, 811113, 811118, 811121, 811122, 811191, 811192, 811198, 448110, 448120, 448130, 448140, 448150, 448190, 448210, 448310, 451110, 451120, 451130, 451140, 451211, *451212*, 442110, 442210, *442291*, 442299, 443141, 443142, 444110, 444120, 444130, 444190, 444210, 444220, 811211, 811212, *811213*, 811219, 811411, 811412, 811420, 811430, 811490
2	Groceries & General Merchandise	445110, 445120, 445210, 445220, 445230, 445291, 445292, 445299, 452210, 452319, 453310
3	Alcohol, Tobacco & Gambling	445310, 722410, 453991, 713210, 713290, 721120
4	Health & Personal Care	446110, 446120, 446130, 446191, 446199, 812111, 812112, 812113, 812191, 812199, *812310*, 812320. 621111, 621112, 621210, 621310, 621320, 621330, 621340, *621391*, 621399, 621410, 621492, 621493, 621498
5	Child Care	624410
6	Family Entertainment & Enrichment	711110, 711120, 711130, 711190, 711211, 711212, 711219, 711310, 712110, 712130, 713110, 713120, 713910, 713920, 713930, 713940, 713950, 713990, 611620, 611630, 611691, 611699, 611710
7	Restaurants	722511, 722513, 722514, 722515

Note: Italicized NAICS codes are those with no observed places in our dataset, but would have been included in the category if such places were observed. The category names are the authors' labeling.

Appendix J: Disaggregated results for visits to automobile, clothing, hobby and sporting goods, and home product stores

Figure J1: Event study specification for visits to automobile, clothing, hobby and sporting goods, and home product stores

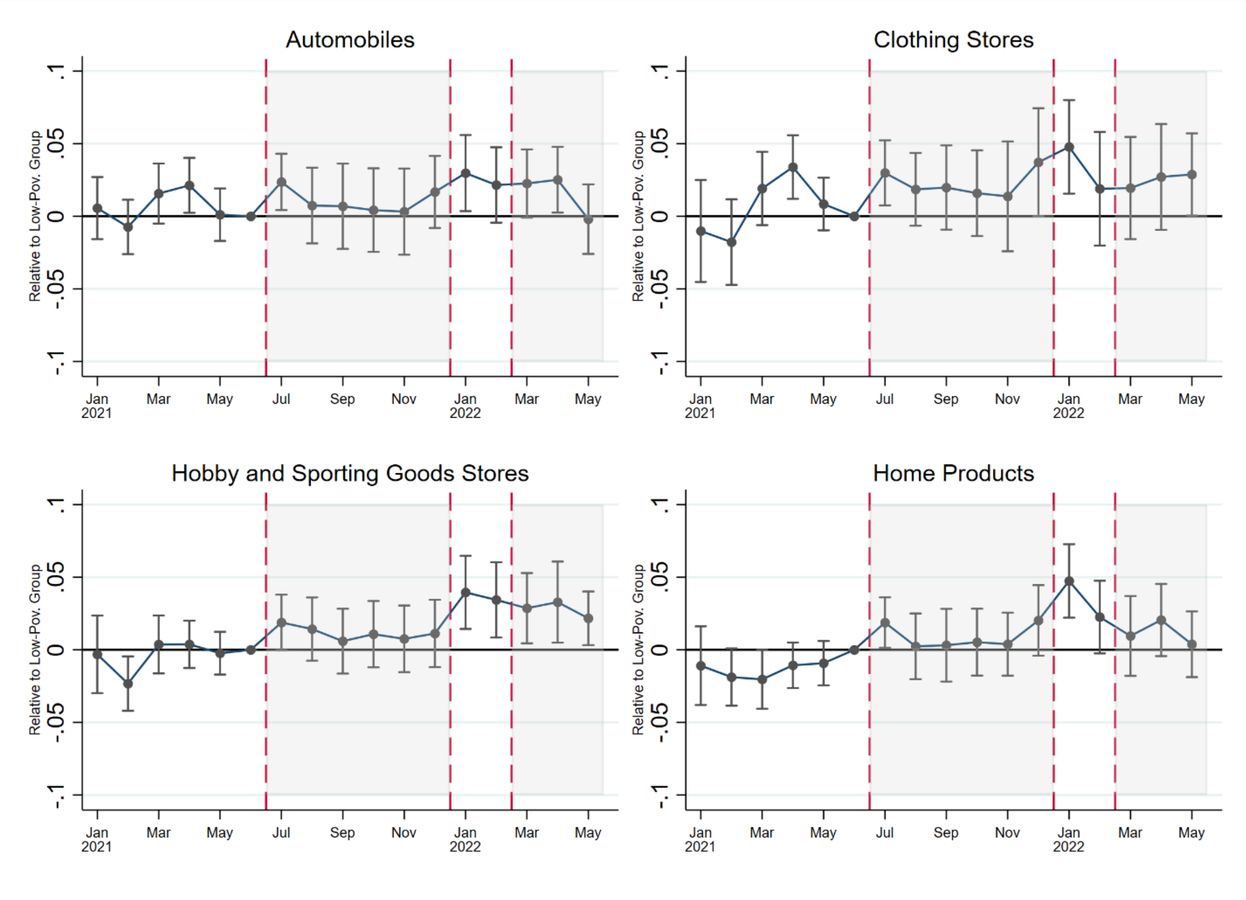

Note: The graphs display the estimated coefficients of an event-study analysis of in-person visits at different establishment types over time, comparing counties in the high and mid-poverty terciles to counties in the lowest-poverty tercile. Estimates are relative to June 2021. The gray, shaded areas represent the monthly and lump-sum CTC treatment periods, respectively. Sample sizes are 43,638 (Automobile), 33,966 (Clothing), 41,735 (Hobby and Sporting Goods), and 46,631 (Home Products). The capped horizontal bars represent 95 percent confidence intervals.
