# Supplementary material for: Consumption responses to an unconditional child allowance in the United States
Source: Nat Hum Behav. 2024 Feb 19;8(4):657–67. doi: 10.1038/s41562-024-01835-6 (PMC11045438; doi:10.1038/s41562-024-01835-6)
Supplement: Supplementary file 2 — Reporting Summary [file 41562_2024_1835_MOESM2_ESM.pdf]

nature portfolio

Corresponding author(s): Zachary Parolin

Last updated by author(s): Oct 25, 2023

Reporting Summary

Nature Portfolio wishes to improve the reproducibility of the work that we publish. This form provides structure for consistency and transparency in reporting. For further information on Nature Portfolio policies, see our Editorial Policies and the Editorial Policy Checklist.

Statistics

For all statistical analyses, confirm that the following items are present in the figure legend, table legend, main text, or Methods section.

n/a	Confirmed	
☐	☒	The exact sample size (*n*) for each experimental group/condition, given as a discrete number and unit of measurement
☐	☒	A statement on whether measurements were taken from distinct samples or whether the same sample was measured repeatedly
☐	☒	The statistical test(s) used AND whether they are one- or two-sided<br>*Only common tests should be described solely by name; describe more complex techniques in the Methods section.*
☐	☒	A description of all covariates tested
☐	☒	A description of any assumptions or corrections, such as tests of normality and adjustment for multiple comparisons
☐	☒	A full description of the statistical parameters including central tendency (e.g. means) or other basic estimates (e.g. regression coefficient) AND variation (e.g. standard deviation) or associated estimates of uncertainty (e.g. confidence intervals)
☐	☒	For null hypothesis testing, the test statistic (e.g. *F*, *t*, *r*) with confidence intervals, effect sizes, degrees of freedom and *P* value noted<br>*Give P values as exact values whenever suitable.*
☒	☐	For Bayesian analysis, information on the choice of priors and Markov chain Monte Carlo settings
☒	☐	For hierarchical and complex designs, identification of the appropriate level for tests and full reporting of outcomes
☒	☐	Estimates of effect sizes (e.g. Cohen's *d*, Pearson's *r*), indicating how they were calculated

Our web collection on statistics for biologists contains articles on many of the points above.

Software and code

Policy information about availability of computer code

Data collection	No software was used for unique data collection. We imported the data from SafeGraph into Stata version 17.
Data analysis	We conduct all analyses using Stata version 17.

For manuscripts utilizing custom algorithms or software that are central to the research but not yet described in published literature, software must be made available to editors and reviewers. We strongly encourage code deposition in a community repository (e.g. GitHub). See the Nature Portfolio guidelines for submitting code & software for further information.

Data

Policy information about availability of data

All manuscripts must include a data availability statement. This statement should provide the following information, where applicable:

- Accession codes, unique identifiers, or web links for publicly available datasets
- A description of any restrictions on data availability
- For clinical datasets or third party data, please ensure that the statement adheres to our policy

We provide the code to replicate our findings in an online repository stored at https://osf.io/gd5wu/. The data that support the findings of this study are available from SafeGraph, a private company that regulates registered users' distribution of its data. For this reason, we cannot publish the underlying SafeGraph dataset online. The mobility and debit/credit card spending data are both available from SafeGraph after registering for data access at https://www.safegraph.com/. The replication code in our online files can convert the raw data into a usable dataset.

Research involving human participants, their data, or biological material

Policy information about studies with human participants or human data. See also policy information about sex, gender (identity/presentation), and sexual orientation and race, ethnicity and racism.

Reporting on sex and gender	Sex- or gender-based analyses are not possible within our analysis, as the mobility and card spending data are aggregated to the place level. We cannot disaggregate consumption patterns by sex or gender.
Reporting on race, ethnicity, or other socially relevant groupings	Race/ethnicity-based analyses are not possible within our analysis, as the mobility and card spending data are aggregated to the place level.
Population characteristics	Our place-based data covers nearly all counties in the United States during 2019 and 2021.
Recruitment	We rely on secondary data and did not recruit participants for the study.
Ethics oversight	The study relies on the use of secondary data that is legally collected, aggregated and anonymized upon collection, and made freely available to academic researchers. Ethics approval was not required.

Note that full information on the approval of the study protocol must also be provided in the manuscript.

Field-specific reporting

Please select the one below that is the best fit for your research. If you are not sure, read the appropriate sections before making your selection.

☐ Life sciences ☒ Behavioural & social sciences ☐ Ecological, evolutionary & environmental sciences

For a reference copy of the document with all sections, see nature.com/documents/nr-reporting-summary-flat.pdf

Behavioural & social sciences study design

All studies must disclose on these points even when the disclosure is negative.

Study description	This study uses quantitative data from SafeGraph to estimate mobility and spending responses to the expansion of the Child Tax Credit in 2021.
Research sample	The SafeGraph sample includes more than 40 million mobile device users across the United States. The SafeGraph data collected is anonymous at point of collection. As detailed in the manuscript, the mobile devices are dispersed randomly across the U.S. and align with Census population counts by county and state. We use the anonymized, aggregated data that SafeGraph provides free-of-charge to researchers. The sample is meant to represent the population of U.S. residents, relevant because this is the group affected by the treatment that we study (the Child Tax Credit expansion).
Sampling strategy	The sample includes mobile devices across the U.S. that agree to (or do not elect not to) share their anonymized location information with third-parties from whom SafeGraph collects data. The SafeGraph data collected is anonymous at point of collection. Data made available for researchers are anonymous and aggregated to the month and point-of-interest (e.g. shops, schools, child care centers, etc.) levels. Samples are non-random and sample sizes are determined based on the full availability of data providers to SafeGraph's aggregation of mobility and spending data.
Data collection	The sample includes mobile devices across the U.S. that agree to (or do not elect not to) share their anonymized location information with third-parties from whom SafeGraph collects data. The SafeGraph data collected is anonymous at point of collection. Data made available for researchers are anonymous and aggregated to the month and point-of-interest (e.g. shops, schools, child care centers, etc.) levels. SafeGraph collects the data from third-party data providers.
Timing	Data collection is continuous among SafeGraph devices. The data made public are aggregates over the course of all days in a given month. The data run from January 1, 2019, to June 1, 2022.
Data exclusions	Individuals without mobile devices or who elect not to share their data with third-party sources are excluded from the SafeGraph data universe. As discussed in the manuscript, non-participants do not appear to bias the data; SafeGraph mobile device counts are strongly and positively correlated with Census population counts across county and state.
Non-participation	Individuals without mobile devices or who elect not to share their data with third-party sources are excluded from the SafeGraph data universe. As discussed in the manuscript, non-participants do not appear to bias the data; SafeGraph mobile device counts are strongly and positively correlated with Census population counts across county and state.
Randomization	Randomization was not necessary for this study. We control through covariates at the county level using data from the U.S. Census Bureau and Opportunity Insights, as detailed in the manuscript.

Reporting for specific materials, systems and methods

We require information from authors about some types of materials, experimental systems and methods used in many studies. Here, indicate whether each material, system or method listed is relevant to your study. If you are not sure if a list item applies to your research, read the appropriate section before selecting a response.

Materials & experimental systems

n/a	Involved in the study
☒	☐ Antibodies
☒	☐ Eukaryotic cell lines
☒	☐ Palaeontology and archaeology
☒	☐ Animals and other organisms
☒	☐ Clinical data
☒	☐ Dual use research of concern
☒	☐ Plants

Methods

n/a	Involved in the study
☒	☐ ChIP-seq
☒	☐ Flow cytometry
☒	☐ MRI-based neuroimaging

Plants

Seed stocks

Report on the source of all seed stocks or other plant material used. If applicable, state the seed stock centre and catalogue number. If plant specimens were collected from the field, describe the collection location, date and sampling procedures.

Novel plant genotypes

Describe the methods by which all novel plant genotypes were produced. This includes those generated by transgenic approaches, gene editing, chemical/radiation-based mutagenesis and hybridization. For transgenic lines, describe the transformation method, the number of independent lines analyzed and the generation upon which experiments were performed. For gene-edited lines, describe the editor used, the endogenous sequence targeted for editing, the targeting guide RNA sequence (if applicable) and how the editor was applied.

Authentication

Describe any authentication procedures for each seed stock used or novel genotype generated. Describe any experiments used to assess the effect of a mutation and, where applicable, how potential secondary effects (e.g. second site T-DNA insertions, mosiacism, off-target gene editing) were examined.
